# Configuration-Level Genetic Interpretation in Neurodevelopmental Disorders: A Single-Center Cohort of 2162 Children in China

**DOI:** 10.3390/genes17091006

**Published:** 2026-08-26

**Authors:** Lingxue Li, Dawei Cheng, Bing Wang, Fan Wu, Xinna Ji, Qian Chen

**Affiliations:** 1 Department of Neurology, Capital Institute of Pediatrics, Chinese Academy of Medical Sciences & Peking Union Medical College, Beijing 100020, China; lilingxue1108@163.com (L.L.); wb17810265082@163.com (B.W.);; 2 Department of Neurology, Capital Center for Children’s Health, Capital Medical University, Capital Institute of Pediatrics, Beijing 100020, Chinajina1227@163.com (X.J.)

**Keywords:** neurodevelopmental disorders, exome sequencing, compound heterozygosity, multilocus molecular diagnosis, mosaicism, uniparental disomy, mitochondrial DNA heteroplasmy

## Abstract

**Background/Objectives:** Compound heterozygosity, multilocus molecular diagnoses, mosaicism, uniparental disomy (UPD), and mitochondrial DNA (mtDNA) heteroplasmy are established configuration-dependent diagnostic categories whose interpretation requires consideration of allelic phase, dosage, parental origin, and tissue distribution. This study evaluated their diagnostic contribution and clinical characteristics in pediatric neurodevelopmental disorders (NDDs). **Methods:** This single-center retrospective cohort study conducted a descriptive cross-phenotypic evaluation of 2162 children in China who underwent clinical genetic testing between 2015 and 2024. The primary analysis focused on confirmed diagnostic configurations, whereas study-defined possibly diagnostic configurations were included only in expanded-set sensitivity and supplementary descriptive analyses. Binary clinical outcomes were adjusted for sex, calendar year of testing, and primary testing strategy. Ascertainment depended on the genetic tests performed for each child rather than systematic screening for all five categories. **Results:** Confirmed diagnostic configurations were identified in 166/1009 (16.5%) children with definitive molecular diagnoses, corresponding to 166/2162 (7.7%) of the full cohort. These comprised 103 compound heterozygous configurations, 24 multilocus molecular diagnoses, 19 mosaic findings, 4 UPD-related configurations, and 16 mtDNA heteroplasmy configurations. An additional 34 children had possibly diagnostic configurations, yielding an expanded configuration set of 200/2162 (9.3%), which was not interpreted as a definitive diagnostic yield. In the confirmed-only analysis, age at onset was lower than in the comparison group (median 1.80 vs. 2.80 years, Bonferroni-adjusted *p* = 0.008), and epilepsy was less frequent [94/166 (56.6%) vs. 1338/1962 (68.2%), Bonferroni-adjusted *p* = 0.035]. **Conclusions:** Confirmed diagnostic configurations involving these five categories accounted for 16.5% of definitive molecular diagnoses and were characterized by a lower frequency of epilepsy at the cohort level. Configuration-level assessment of allelic phase, dosage, multilocus contribution, parental origin, mosaic fraction, and tissue context may improve diagnostic completeness and recurrence-risk counseling.

## 1. Introduction

Exome sequencing (ES) is an essential approach to the genetic diagnosis of neurodevelopmental disorders (NDDs) in children, with reported diagnostic rates of approximately 30–50% [1,2,3]. However, the clinical interpretation of a genetic finding may depend not only on the pathogenicity of an individual variant but also on its allelic phase, dosage context, multilocus contribution, parental origin, variant allele fraction, and tissue distribution. These configuration-level features can influence the interpretation of phenotype, inheritance, recurrence risk, and tissue specificity.

Compound heterozygosity, multilocus molecular diagnoses, mosaicism, uniparental disomy (UPD), and mitochondrial DNA (mtDNA) heteroplasmy are established configuration-dependent diagnostic categories relevant to the genetic diagnosis of NDDs. Compound heterozygous diagnoses require two disease-associated alleles in trans; in some cases, a copy-number variant (CNV) constitutes the second allele [4,5]. Multilocus molecular diagnoses arise when two or more independent genetic conditions jointly contribute to a blended or composite phenotype [6,7]. Mosaic findings require consideration of variant allele fraction, tested tissue, and parental origin, and parental mosaicism may alter recurrence-risk estimates [8,9]. UPD may cause disease through imprinting abnormalities or the unmasking of a recessive allele [10]. For mtDNA variants, clinical relevance may vary with heteroplasmy level and the tissue analyzed [11,12].

**Figure 1:**
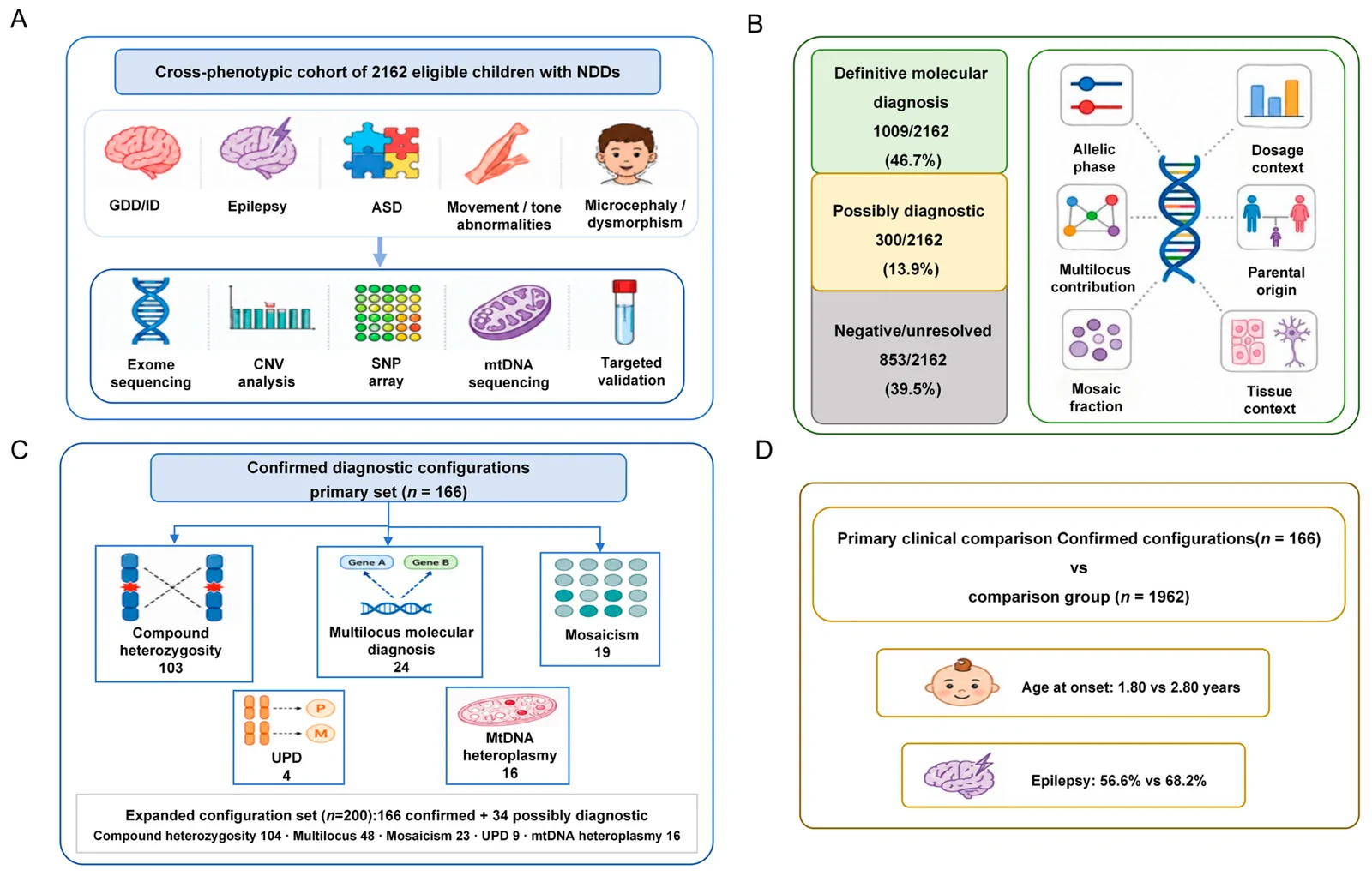
Study workflow. (**A**) Study cohort, eligible phenotypes, and exome sequencing (ES)-centered testing workflow. (**B**) Patient-level diagnostic classification and key interpretive dimensions. Green, yellow, and gray indicate definitive molecular diagnosis, possibly diagnostic findings, and negative/unresolved findings, respectively. (**C**) Distribution of the 166 confirmed diagnostic configurations across the five prespecified configuration categories; the expanded set of 200 configurations, comprising 166 confirmed and 34 possibly diagnostic configurations, was used for secondary descriptive and sensitivity analyses. (**D**) Primary clinical comparison between children with confirmed diagnostic configurations and the comparison group.

Previous pediatric ES studies have primarily focused on overall diagnostic yield and clinical utility [1,2,3], whereas the five configuration categories described above have generally been investigated separately. Our previous phenotype-specific studies focused on the genetic landscape and diagnostic yield of pediatric epilepsy and global developmental delay/intellectual disability (GDD/ID) [13,14]. Although compound heterozygosity is routinely assessed during ES interpretation, it has rarely been considered together with multilocus molecular diagnoses, mosaicism, UPD, and mtDNA heteroplasmy within a unified analytical framework. We therefore conducted a descriptive cross-phenotypic evaluation of these five prespecified categories in a cohort of 2162 children in China who underwent ES-centered clinical genetic testing. This cross-phenotypic approach was chosen because the categories are defined by diagnostic architecture rather than by a single neurodevelopmental phenotype. Relevant configurations were classified as confirmed diagnostic, possibly diagnostic, or candidate. The primary aim was to quantify the contribution of confirmed diagnostic configurations among definitive molecular diagnoses and to characterize the clinical features of children in whom they were identified (Figure 1).

## 2. Materials and Methods

### 2.1. Study Population

This single-center retrospective cohort study included 2162 children with NDDs who were evaluated at a tertiary pediatric center in Beijing, China; underwent clinical genetic testing between January 2015 and December 2024, and met the prespecified eligibility criteria. Additional details regarding ethics approval and institutional naming are provided in Supplementary Methods S1. The cohort was cross-phenotypic and was not restricted to a single province or region of residence.

Inclusion criteria were as follows. (1) At least one eligible neurodevelopmental phenotype, including GDD, ID, epilepsy occurring in an NDD context, autism spectrum disorder (ASD), movement disorders, abnormal muscle tone, microcephaly, facial dysmorphism, or related neurodevelopmental or syndromic features. (2) Clinical genetic testing within an exome sequencing (ES)-centered diagnostic workflow. (3) Sufficient clinical and genetic-testing data to determine patient-level diagnostic status and, when a finding within one of the five prespecified configuration categories was identified, to assign its configuration-level classification. Exclusion criteria were: (1) a clear acquired cause, including trauma, central nervous system infection, cerebrovascular disease, perinatal brain injury, or another defined acquired insult; (2) isolated provoked seizures without an additional eligible neurodevelopmental phenotype; and (3) isolated non-neurodevelopmental findings.

Some participants had been included in our previously reported phenotype-specific cohorts of epilepsy (*n* = 1109) and GDD/ID (*n* = 1024) [13,14]. In the present cohort, 730 children (33.8%) overlapped only with the previous epilepsy cohort, 578 (26.7%) only with the previous GDD/ID cohort, and 269 (12.4%) with both cohorts. The remaining 585 children (27.1%) had not been included in either previous study, and 1577/2162 (72.9%) had appeared in at least one previous publication. The present cohort was not constructed by simply merging data from the two previous cohorts. Instead, all eligible clinical genetic-testing records were independently re-screened according to the current eligibility and data-sufficiency criteria and deduplicated at the patient level. The previous epilepsy study focused on diagnostic yield, gene spectrum, and clinical factors associated with molecular diagnosis, whereas the GDD/ID study focused on diagnostic yield, genetic landscape, genotype–phenotype associations, and functional characteristics of disease-associated genes. In contrast, the present study used configuration status rather than a specific neurodevelopmental phenotype as the analytical framework and evaluated the diagnostic contribution and clinical and configuration-specific characteristics of five prespecified configuration-dependent diagnostic categories across the NDD cohort.

### 2.2. Genetic Testing and Patient-Level Diagnostic Classification

Clinical genetic testing was centered on trio or proband-only ES. Duo ES (proband and one parent) and targeted-panel sequencing were also used in a subset of children. When clinically indicated, additional or complementary analyses included ES read-depth-based CNV calling, chromosomal microarray or single-nucleotide polymorphism (SNP)-array analysis, multiplex ligation-dependent probe amplification (MLPA), full-length mtDNA next-generation sequencing (NGS), and targeted validation of selected mosaic, UPD, CNV, or mtDNA findings.

The genetic testing workflow evolved over the study period. Targeted-panel sequencing was used predominantly during the earlier study years, whereas ES became the principal testing strategy from 2018 onward, with trio ES accounting for approximately three-quarters of testing in both 2018–2020 and 2021–2024. The use of complementary SNP-array, MLPA, and full-length mtDNA NGS also varied over time according to clinical indication. Annual distributions of testing strategies are shown in Figure S2.

Sequence variants were interpreted according to ACMG/AMP standards, and CNVs according to ACMG/ClinGen standards [15,16]. For analytical purposes, the primary testing strategy was categorized as trio ES, non-trio ES (proband-only or duo ES), or targeted-panel/other testing.

At the patient level, a definitive molecular diagnosis required at least one phenotype-concordant finding with diagnostic-level evidence. This category included pathogenic or likely pathogenic (P/LP) variants with disease-consistent inheritance and zygosity, diagnostic CNVs, confirmed UPD- or imprinting-related diagnoses, and pathogenic or likely pathogenic mtDNA variants. Possibly diagnostic status was assigned to phenotype-concordant findings that did not meet definitive criteria. These included a variant of uncertain significance (VUS) in a compatible autosomal-dominant or X-linked gene; a P/LP variant plus a VUS or VUS plus VUS in a compatible autosomal-recessive gene; a phenotype-compatible CNV classified as a VUS; or other phenotype-concordant findings with incomplete diagnostic evidence. Children without a phenotype-relevant finding meeting either threshold were classified as negative or unresolved. The term “possibly diagnostic” was a study-defined designation used at both the patient and configuration levels and was distinct from standard ACMG/AMP variant classification.

### 2.3. Configuration-Specific Classification

Findings involving autosomal-recessive compound heterozygosity, multilocus molecular diagnoses, mosaicism, UPD, and mtDNA heteroplasmy were subsequently evaluated at the configuration level. Each configuration was classified as confirmed diagnostic, possibly diagnostic, or candidate according to the operational framework described in Supplementary Methods S2, with category-specific criteria detailed in Supplementary Methods S2.1–S2.5. For multilocus molecular diagnoses, confirmed diagnostic status required at least two independent molecular components, each meeting diagnostic-level evidence; configurations containing one or more components below this threshold were classified as possibly diagnostic.

Ascertainment of the five configuration categories depended on the genetic tests actually performed for each child. Participants were not required to have undergone dedicated testing capable of systematically detecting or excluding all five categories. Accordingly, the absence of a given configuration was not interpreted as evidence that it had been systematically excluded when the relevant testing was not performed.

The primary analytical set was restricted to confirmed diagnostic configurations. Confirmed and possibly diagnostic configurations were combined only in an expanded configuration set used for sensitivity and supplementary descriptive analyses, whereas candidate configurations were retained for descriptive reporting only. Primary clinical comparisons were conducted at the patient level between children with confirmed diagnostic configurations and a comparison group comprising children without confirmed or possibly diagnostic configurations in the five prespecified categories.

Clinical variables included age at onset, sex, GDD/ID, epilepsy, ASD, abnormal muscle tone, facial dysmorphism, microcephaly, and family history. Family history was defined as a relevant neurological or neurodevelopmental history within three generations.

Because individual clinical phenotypes were not mutually exclusive, their frequencies were calculated independently. To further characterize the distribution of GDD/ID and epilepsy, patients were classified into four mutually exclusive categories: GDD/ID without epilepsy, epilepsy without GDD/ID, both phenotypes, and neither phenotype. Other clinical features were permitted within each category. For supplementary descriptive analysis, genes represented in the expanded configuration set were mapped to the recorded clinical phenotype groups, yielding overlapping phenotype-specific gene sets. Principal biological functions/pathways represented within each gene set were summarized descriptively from established gene/protein functions; no formal statistical enrichment analysis was performed.

Origin and tissue context were recorded when available. Configuration-specific information included allelic phase and second-allele dosage for compound heterozygosity; inheritance of multilocus components; origin and tissue distribution of mosaicism; parental origin of UPD; and maternal inheritance, tested tissue, and heteroplasmy level for mtDNA findings.

### 2.4. Statistical Analysis

Continuous variables were summarized as medians and interquartile ranges. Age at onset was compared between two groups using the Mann–Whitney *U* test and across the five confirmed diagnostic configuration categories using the Kruskal–Wallis test as an exploratory analysis. Categorical variables were presented as counts and percentages and compared using the chi-square test or Fisher’s exact test, as appropriate.

Binary clinical outcomes were analyzed using multivariable logistic regression adjusted for sex, calendar year of testing, and primary testing strategy. The model for sex excluded sex as a covariate. Observations with missing outcome or covariate data were excluded from the corresponding analysis; complete-case analysis was used for the covariates included in each regression model.

Family history, primary testing strategy, and the four-category GDD/ID–epilepsy distribution were reported descriptively and were not included among the eight prespecified clinical comparisons, across which Bonferroni correction was applied. The primary clinical comparisons were repeated using the expanded configuration set as a sensitivity analysis.

To investigate potential temporal bias, the study period was grouped into 2015–2017, 2018–2020, and 2021–2024, and differences in the proportion of children with confirmed diagnostic configurations across testing periods were evaluated using logistic regression adjusted for primary testing strategy. All *p* values were two-sided, and adjusted *p* values < 0.05 were considered statistically significant. Analyses were performed using R version 4.5.1 (R Foundation for Statistical Computing, Vienna, Austria) and GraphPad Prism version 10.0 (GraphPad Software, Boston, MA, USA).

## 3. Results

### 3.1. Cohort Overview and Diagnostic Contribution

The analytic cohort comprised 2162 eligible children. Residential region was available for 2014/2162 children (93.2%). Of these, 1863/2014 (92.5%) resided in North China, 89/2014 (4.4%) in Northeast China, 30/2014 (1.5%) in Northwest China, 22/2014 (1.1%) in East China, and 10/2014 (0.5%) in Central China (Figure S1). At the patient level, 1009 children (46.7%) received a definitive molecular diagnosis, 300 (13.9%) had possibly diagnostic findings, and 853 (39.5%) were negative or unresolved (Figure 1B). Sex was recorded as male in 1285 children (59.4%) and female in 836 (38.7%), with missing or unknown information for 41 (1.9%). Among the 2104 children with available age-at-onset data, the median age at onset was 2.80 years (IQR, 1.00–5.80).

Confirmed diagnostic configurations in the five prespecified categories were identified in 166/1009 children with definitive molecular diagnoses (16.5%), corresponding to 166/2162 (7.7%) of the full cohort. An additional 34 children had possible diagnostic configurations, yielding an expanded configuration set of 200 children (9.3% of the full cohort) for secondary and sensitivity analyses. Three candidate-only mosaic findings were retained for descriptive reporting only.

**Figure 2:**
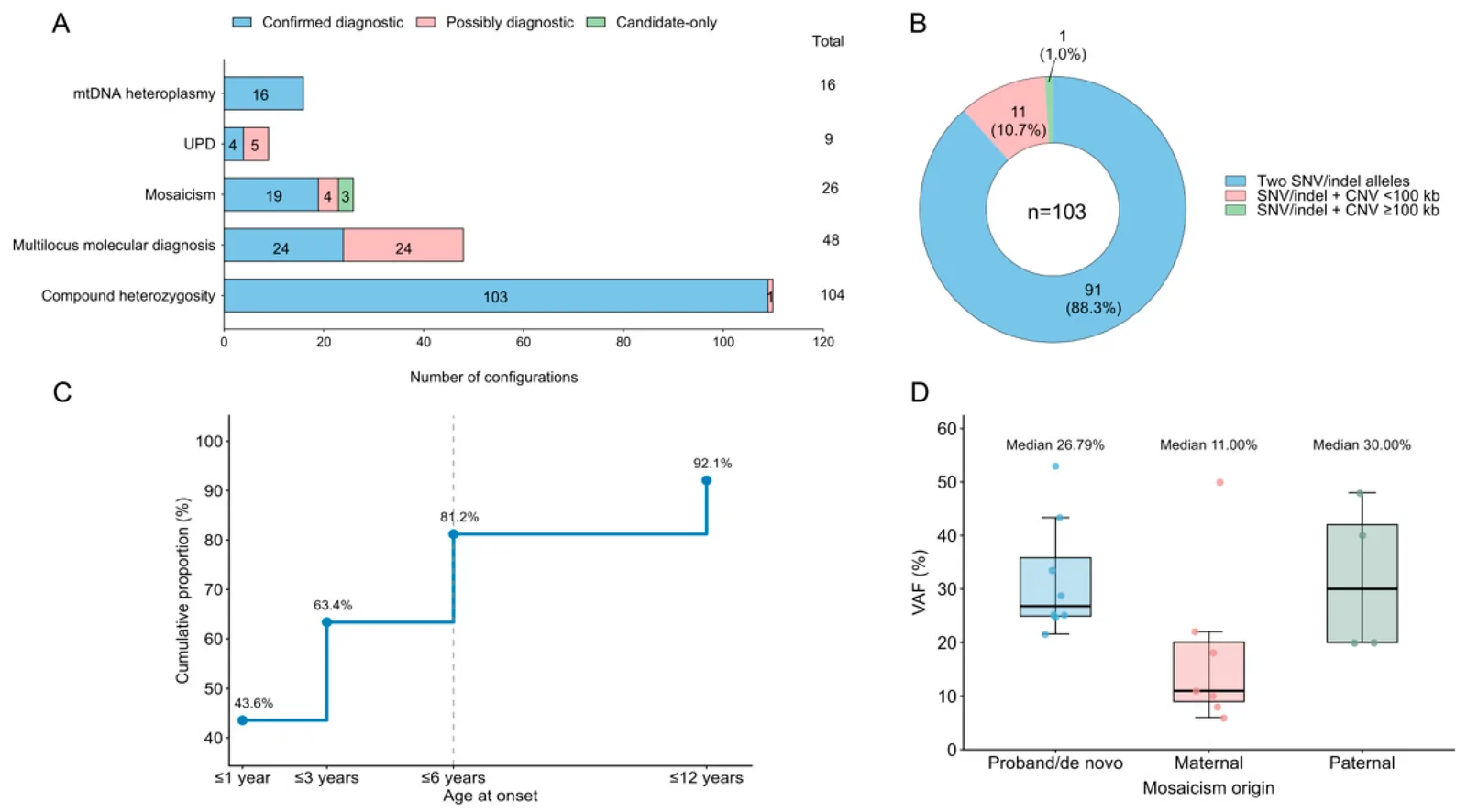
Configuration-Level Characteristics. (**A**) Distribution of all 203 identified configurations across the five configuration categories and evidence tiers, comprising 166 confirmed diagnostic configurations, 34 possibly diagnostic configurations, and 3 candidate-only mosaic findings. (**B**) Allelic composition of confirmed compound heterozygous configurations (*n* = 103). (**C**) Cumulative age-at-onset distribution in confirmed compound heterozygosity (*n* = 101 with available age-at-onset data). The dashed vertical line indicates the 6-year age threshold. (**D**) VAF (variant allele fraction) distribution by mosaicism origin (*n* = 19).

The 166 confirmed diagnostic configurations comprised 103 compound heterozygous configurations, 24 multilocus molecular diagnoses, 19 mosaic findings, 16 mtDNA heteroplasmy findings, and 4 UPD-related configurations. Figure 2A provides a descriptive overview of all 203 identified configurations across evidence tiers, comprising 166 confirmed diagnostic, 34 possibly diagnostic, and 3 candidate-only findings. Patient-level genotypic and diagnostic characteristics of the expanded configuration set are provided in Table S1, and phenotype-specific gene sets and their principal biological functions are summarized separately for confirmed and possibly diagnostic configurations in Table S2.

Because testing strategies changed over the study period, we additionally examined confirmed diagnostic configurations across three testing eras. Confirmed diagnostic configurations were identified in 31/495 children (6.3%) tested in 2015–2017, 76/735 (10.3%) in 2018–2020, and 59/932 (6.3%) in 2021–2024 (chi-square *p* = 0.004). After adjustment for primary testing strategy, neither 2018–2020 (adjusted OR 1.28, 95% CI 0.67–2.45, *p* = 0.449) nor 2021–2024 (adjusted OR 0.77, 95% CI 0.40–1.48, *p* = 0.431) differed significantly from 2015–2017 (Table S3).

### 3.2. Clinical Characteristics by Configuration Status

The primary comparison included 166 children with confirmed diagnostic configurations and 1962 children in the comparison group. Children with confirmed diagnostic configurations had an earlier age at onset than the comparison group (median, 1.80 years [IQR 0.58–5.00, *n* = 162] vs. 2.80 years [IQR 1.00–5.80, *n* = 1909], Bonferroni-adjusted *p* = 0.008), corresponding to a 1.0-year difference in the group medians. In an exploratory comparison across the five confirmed diagnostic configuration categories, median age at onset was 1.57 years for compound heterozygosity, 1.65 years for multilocus molecular diagnoses, 1.25 years for mosaicism, 2.82 years for UPD, and 3.10 years for mtDNA heteroplasmy. The overall difference across the five categories was not statistically significant (*p* = 0.160, Table S4).

Children with confirmed diagnostic configurations also had a lower frequency of epilepsy [94/166 (56.6%) vs. 1338/1962 (68.2%), adjusted OR 0.62, 95% CI 0.44–0.86, Bonferroni-adjusted *p* = 0.035]. A positive family history was recorded in 19/166 (11.4%) children with confirmed diagnostic configurations and 161/1962 (8.2%) children in the comparison group (*p* = 0.150). No other prespecified clinical difference remained significant after Bonferroni correction (Table 1).

Among children with confirmed diagnostic configurations, 63/166 (38.0%) had GDD/ID without epilepsy, 75/166 (45.2%) had epilepsy without GDD/ID, 19/166 (11.4%) had both phenotypes, and 9/166 (5.4%) had neither phenotype. The corresponding proportions in the comparison group were 515/1962 (26.2%), 1099/1962 (56.0%), 239/1962 (12.2%), and 109/1962 (5.6%), respectively. The overall distribution of the four categories differed between groups (*p* = 0.011, Table 1).

Expanded-set sensitivity analyses are presented in Table S5. The differences in age at onset and epilepsy remained significant, while the difference in GDD/ID reached statistical significance after possible diagnostic configurations were included.

### 3.3. Compound Heterozygosity and CNV Second Alleles

The primary analysis included 103 confirmed diagnostic compound heterozygous configurations. Among these configurations, 91 (88.3%) involved two SNV/indel alleles, 11 (10.7%) involved an SNV/indel and a CNV < 100 kb, and 1 (1.0%) involved an SNV/indel and a CNV ≥ 100 kb. Overall, 12/103 configurations (11.7%) involved a CNV as the second allele (Figure 2B).

Age-at-onset data were available for 101 of the 103 children. Onset had occurred by age 1 year in 44 children (43.6%), by age 3 years in 64 (63.4%), by age 6 years in 82 (81.2%), and by age 12 years in 93 (92.1%) (Figure 2C). Expanded-set results are provided in Table S6.

### 3.4. Multilocus Molecular Diagnoses

The primary analysis included 24 confirmed diagnostic multilocus molecular diagnoses. At least one de novo molecular component was identified in 22/24 configurations (91.7%), and at least one component involved a CNV or exon-level copy-number alteration in 12/24 (50.0%).

Age-at-onset data were available for all 24 children. Onset occurred before age 2 years in 13 children (54.2%) and before age 5 years in 20 (83.3%). No single recurrent gene-pair combination predominated. Expanded-set results are provided in Table S7.

### 3.5. Mosaicism and Parental Origin

The primary analysis included 19 confirmed diagnostic mosaic findings. Origin was classified as proband/de novo in 8 findings (42.1%), maternal in 7 (36.8%), and paternal in 4 (21.1%).

Of these findings, 17/19 (89.5%) were assessed by ES and 2/19 (10.5%) by targeted next-generation sequencing panels; peripheral blood-derived DNA was analyzed in 18/19 (94.7%), with one tissue-restricted event detected in affected skin or mucosal tissue. Sequencing depth was available for all 19 findings (median, 138.42×; range, 90.68×–177.90×).

VAF data were available for all 19 findings. The median VAF was 26.79% for the 8 proband/de novo findings, 11.00% for the 7 maternal mosaic findings, and 30.00% for the 4 paternal mosaic findings. When maternal and paternal findings were combined, the median VAF among the 11 confirmed parental mosaic findings was 20.00%, and 8/11 (72.7%) had a VAF < 25% (Figure 2D). Expanded-set and candidate-only findings are provided in Table S8.

### 3.6. UPD and mtDNA Heteroplasmy

The primary analysis included four confirmed diagnostic UPD-related configurations. These comprised two paternal chromosome 15 UPD findings consistent with Angelman syndrome, one maternal chromosome 15 UPD finding consistent with Prader–Willi syndrome, and one X-chromosome isodisomy finding retained with configuration-specific annotation. Expanded-set results are provided in Table S9.

The mtDNA heteroplasmy category included 16 confirmed diagnostic configurations. All 16 configurations were assessed using full-length mitochondrial genome next-generation sequencing. Eleven findings (68.8%) were maternally inherited, whereas five (31.3%) were classified as de novo or not detected in available maternal samples. Paired blood–urine heteroplasmy measurements were available for four findings. Three of the four paired sample sets showed an absolute blood–urine heteroplasmy difference >10%, whereas one showed a difference ≤ 10%. These comparisons were descriptive because of the limited number of paired samples (Table S10).

## 4. Discussion

In this cross-phenotypic cohort of 2162 children with NDDs, 1009 children received a definitive molecular diagnosis. Confirmed diagnostic configurations involving one of the five prespecified configuration categories were identified in 166 of 1009 definitively diagnosed children (16.5%), equivalent to approximately one in six. Genome-wide and follow-up diagnostic studies have shown that additional coding, structural, and non-coding diagnoses may be identified beyond conventional ES-centered testing [17,18,19]. Rather than implying that these established categories are overlooked by conventional variant interpretation, the present study evaluated their diagnostic contribution within a unified configuration-level framework that distinguished patient-level diagnostic status from configuration-level classification. These findings indicate that a meaningful proportion of pediatric molecular diagnoses requires consideration of allelic phase, dosage, multilocus contribution, parental origin, and tissue context beyond isolated-variant classification alone.

Children with confirmed diagnostic configurations had an age at onset approximately 1 year earlier at the median and a lower frequency of epilepsy compared with the comparison group. However, age at onset did not differ significantly across the five confirmed diagnostic configuration categories in the exploratory analysis. The analysis of the four mutually exclusive GDD/ID–epilepsy categories showed that the difference in their overall distribution was characterized mainly by a higher proportion of GDD/ID without epilepsy and a lower proportion of epilepsy without GDD/ID, whereas the proportions of children with both phenotypes or neither phenotype were similar between groups. Clinical and diagnostic profiles vary across pediatric NDD and epilepsy cohorts because of differences in referral patterns, phenotype composition, and testing strategies [13,14]. Although the binary clinical comparisons were adjusted for sex, calendar year of testing, and primary testing strategy, residual ascertainment bias remains possible, and the lower frequency of epilepsy may partly reflect differences in cohort composition. Because the five configuration categories are biologically and diagnostically heterogeneous, these findings should be interpreted as cohort-level differences in phenotype composition rather than causal or configuration-specific associations or individual-level predictors.

In the confirmed diagnostic compound heterozygosity group, 11.7% of configurations involved a CNV as the second allele, with most of these CNVs smaller than 100 kb. Previous studies have demonstrated that CNVs may constitute a pathogenic allele in autosomal-recessive disorders and that clinically relevant exon-level or other small CNVs can be detected through ES read-depth analysis [4,5]. These findings support integrated sequence and copy-number analysis, including targeted CNV validation when indicated, particularly in cases in which only one phenotype-compatible SNV/indel is initially identified in an autosomal-recessive disease gene. The observed proportion is specific to the compound heterozygous configurations identified in this cohort and should not be extrapolated to all autosomal-recessive diagnoses.

Multilocus molecular diagnoses constituted the second-largest configuration category. Previous diagnostic studies have shown that pathogenic variation at two or more loci can produce blended phenotypes or explain clinical features extending beyond an initial molecular diagnosis [6,7]. Additional pathogenic variants may also have independent implications in individuals with an already established genetic disorder [20]. Within the confirmed diagnostic multilocus group, 91.7% involved at least one de novo component and 50.0% included a CNV or exon-level copy-number alteration. The absence of a predominant recurrent gene pair suggests that multilocus interpretation generally requires case-specific assessment across variant classes and inheritance patterns. Consequently, an initial molecular diagnosis should not automatically end genomic evaluation when the phenotype remains broader, more severe, or only partially explained. Each molecular component may have distinct inheritance and recurrence-risk implications.

Parental mosaicism highlights the importance of variant origin in recurrence-risk assessment. Within the confirmed parental mosaic group, 72.7% of findings had a VAF below 25%. The median VAF was 20.00% for parental mosaic findings compared with 26.79% for proband/de novo mosaic findings. Multi-tissue and high-sensitivity studies have shown that occult parental mosaicism can substantially modify recurrence-risk estimates following an apparently de novo diagnosis [21,22]. Genome-wide phasing and deep-sequencing studies further indicate that parental origin and tissue distribution of mosaic variants are important considerations in recurrence-risk assessment [23,24,25]. These findings support a low threshold for reviewing parental sequencing data and performing targeted high-depth validation when an apparently de novo variant has important reproductive implications, or when familial recurrence or an atypical inheritance pattern is observed.

UPD and mtDNA heteroplasmy further demonstrate the need for configuration-specific confirmation. UPD can be detected using trio genotypes and exome-derived homozygosity patterns, but its clinical interpretation depends on parental origin, isodisomy, imprinting, or the unmasking of a recessive allele [26,27]. Homozygosity or a UPD-like pattern alone is therefore insufficient to establish a UPD-related diagnosis. For mtDNA variants, interpretation requires consideration of heteroplasmy level, maternal inheritance, threshold effects, and the tissue analyzed [28,29,30]. Paired blood–urine heteroplasmy measurements were available for 25.0% of the confirmed mtDNA configurations, and 75.0% of these paired measurements showed an absolute cross-tissue difference greater than 10%. This finding supports the relevance of tissue context but is insufficient to define a clinically meaningful threshold or determine the optimal diagnostic tissue.

Several limitations should be considered. First, this retrospective single-center cohort spanned a 10-year period during which testing workflows evolved. Although the proportion of children with confirmed diagnostic configurations did not differ significantly between the later testing eras and 2015–2017 after adjustment for primary testing strategy, residual temporal and ascertainment bias cannot be excluded, particularly for configuration categories requiring specialized or high-sensitivity testing. Trio sequencing, CNV analyses, SNP-array analyses, parental deep validation, mtDNA sequencing, and multi-tissue testing were not performed uniformly. Low-level, gonadal, and tissue-restricted mosaicism and other configuration-specific findings may therefore have been missed. Accordingly, the observed category frequencies represent configurations identified within this clinically tested cohort and should not be interpreted as prevalence estimates based on systematic screening. Because an unaffected control population was not included, the present study cannot assess population-level enrichment or relative disease risk associated with these configuration categories. Second, phenotypic documentation, evidence completeness, and data availability varied across patients. The clinical comparisons may have been influenced by referral and testing selection, and the UPD and mtDNA groups were small. Possibly diagnostic configurations were excluded from all primary clinical and configuration-specific analyses and were retained only in expanded-set sensitivity and supplementary descriptive analyses. Their classification may change with additional segregation, functional, variant-level, or gene–disease evidence. Third, the substantial overlap with our previously reported phenotype-specific epilepsy and GDD/ID cohorts should be considered when comparing findings across publications, although the present study addressed a distinct configuration-level question.

## 5. Conclusions

In this cohort of 2162 children with NDDs, confirmed diagnostic configurations involving the five prespecified categories accounted for 16.5% of definitive molecular diagnoses. Configuration-level assessment of allelic phase, dosage, multilocus contribution, parental origin, mosaic fraction, and tissue context may improve the completeness of molecular interpretation and recurrence-risk counseling. Multicenter studies using standardized testing and validation strategies in consecutively ascertained pediatric NDD cohorts undergoing clinical genetic testing are needed to assess the generalizability and clinical utility of this configuration-level approach.

## Figures and Tables

**Table 1:** Clinical Characteristics According to Confirmed Diagnostic Configuration Status.

Variable	Confirmed Diagnostic Configuration Group (*N* = 166)	Comparison Group (*N* = 1962)	Crude OR (95% CI)	Adjusted OR (95% CI)	*p* Value	Bonferroni-Adjusted *p* Value
Demographic and testing characteristics						
Age at onset, years, median (IQR)	1.80 (0.58–5.00) [*N* = 162]	2.80 (1.00–5.80) [*N* = 1909]		—	<0.001	0.008
Male sex, *n*/*N* (%)	102/166 (61.4%)	1164/1921 (60.6%)	1.04 (0.75–1.44)	1.03 (0.74–1.43)	0.855	1
Positive family history, *n*/*N* (%)	19/166 (11.4%)	161/1962 (8.2%)	1.45 (0.87–2.39)	—	0.150	—
Primary testing strategy, *n*/*N* (%)			—	—	<0.001 ^c^	—
Trio ES	140/166 (84.3%)	1229/1962 (62.6%)	—	—	—	—
Non-trio ES	4/166 (2.4%)	367/1962 (18.7%)	—	—	—	—
Targeted-panel/other testing	22/166 (13.3%)	366/1962 (18.7%)	—	—	—	—
Individual clinical phenotypes, *n*/*N* (%) ^a^						
GDD/ID	82/166 (49.4%)	754/1962 (38.4%)	1.56 (1.14–2.15)	1.48 (1.06–2.05)	0.02	0.161
Epilepsy	94/166 (56.6%)	1338/1962 (68.2%)	0.61 (0.44–0.84)	0.62 (0.44–0.86)	0.004	0.035
ASD	6/166 (3.6%)	65/1962 (3.3%)	1.09 (0.47–2.56)	0.98 (0.41–2.32)	0.965	1
Abnormal muscle tone	26/166 (15.7%)	185/1962 (9.4%)	1.78 (1.14–2.78)	1.75 (1.10–2.76)	0.017	0.138
Facial dysmorphism	6/166 (3.6%)	95/1962 (4.8%)	0.74 (0.32–1.71)	0.75 (0.32–1.76)	0.508	1
Microcephaly	7/166 (4.2%)	58/1962 (3.0%)	1.45 (0.65–3.22)	1.34 (0.59–3.06)	0.485	1
Distribution of GDD/ID and epilepsy, *n*/*N* (%) ^b^			—	—	0.011 ^c^	—
GDD/ID without epilepsy	63/166 (38.0%)	515/1962 (26.2%)	—	—	—	—
Epilepsy without GDD/ID	75/166 (45.2%)	1099/1962 (56.0%)	—	—	—	—
Both GDD/ID and epilepsy	19/166 (11.4%)	239/1962 (12.2%)	—	—	—	—
Neither GDD/ID nor epilepsy	9/166 (5.4%)	109/1962 (5.6%)	—	—	—	—

Data are presented as *n*/*N* (%) or median (IQR). Age at onset was compared using the Mann–Whitney *U* test. Crude ORs were estimated by univariable logistic regression, and adjusted ORs by multivariable logistic regression controlling for sex, calendar year of testing, and primary testing strategy; the sex model omitted sex as a covariate. Bonferroni correction was applied across the eight prespecified clinical comparisons. Family history, primary testing strategy, and the four-category GDD/ID–epilepsy distribution were descriptive and were not included in the Bonferroni correction. ^a^ Individual clinical phenotypes were not mutually exclusive. ^b^ The four GDD/ID–epilepsy categories were mutually exclusive. ^c^ Overall chi-square *p* value. ASD, autism spectrum disorder; CI, confidence interval; ES, exome sequencing; GDD/ID, global developmental delay/intellectual disability; IQR, interquartile range; OR, odds ratio.

## Data Availability

Aggregate data supporting the findings of this study are included in the article and its Supplementary Materials. De-identified individual-level data and the R scripts used for the statistical analyses are available from the corresponding author upon reasonable request, subject to institutional and ethical approval.

## Supplementary Materials

The following supporting information can be downloaded at https://www.mdpi.com/article/10.3390/genes17091006/s1, Supplementary Methods S1. Ethics Approval and Institutional Naming; Supplementary Methods S2. Operational Classification of the Five Prespecified Configuration Categories; Supplementary Methods S2.1. Autosomal-Recessive Compound Heterozygosity; Supplementary Methods S2.2. Multilocus Molecular Diagnosis; Supplementary Methods S2.3. Mosaicism and Parental Mosaicism; Supplementary Methods S2.4. Uniparental Disomy; Supplementary Methods S2.5. Mitochondrial DNA Heteroplasmy; Table S1. Patient-Level Genotypic and Diagnostic Characteristics of the Expanded Configuration Set; Table S2. Clinical Phenotype–Gene Set Mapping and Principal Biological Functions Across Confirmed and Possibly Diagnostic Configurations; Table S3. Temporal Sensitivity Analysis of Confirmed Diagnostic Configuration Contribution; Table S4. Age at Onset Across Confirmed Diagnostic Configuration Categories; Table S5. Sensitivity Analysis Using the Expanded Configuration Set; Table S6. Expanded-Set Compound Heterozygous Configurations; Table S7. Expanded-Set Multilocus Molecular Diagnoses; Table S8. Expanded-Set Mosaic Findings and Candidate-Only Supplementary Records; Table S9. Expanded-Set UPD-Related Configurations; Table S10. Confirmed Diagnostic mtDNA Heteroplasmy Configurations; Figure S1. Geographic Distribution of the Study Cohort; Figure S2. Evolution of Genetic Testing Strategies, 2015–2024.

## Abbreviations

ASD	Autism spectrum disorder
CI	Confidence interval
CNV	Copy-number variant
ES	Exome sequencing
GDD/ID	Global developmental delay/intellectual disability
IQR	Interquartile range
mtDNA	Mitochondrial DNA
NDD	Neurodevelopmental disorder
OR	Odds ratio
P/LP	Pathogenic or likely pathogenic
SNV/indel	Single-nucleotide variant or insertion/deletion
UPD	Uniparental disomy
VAF	Variant allele fraction
VUS	Variant of uncertain significance
