# Supplementary material for: Configuration-Level Genetic Interpretation in Neurodevelopmental Disorders: A Single-Center Cohort of 2162 Children in China
Source: Genes (Basel). 2026 Aug 26;17(9):1006. doi: 10.3390/genes17091006 (PMC13606647; doi:10.3390/genes17091006)
Supplement: Supplementary file 1 [file genes-17-01006-s001.zip › genes-4487312-supplementary.pdf]

Configuration-Level Genetic Interpretation in Neurodevelopmental Disorders: A Single-Center Cohort of 2162 Children in China

Supplementary Methods

Supplementary Methods S1. Ethics Approval and Institutional Naming
Supplementary Methods S2. Operational Classification of the Five Prespecified Configuration Categories
Supplementary Methods S2.1. Autosomal-Recessive Compound Heterozygosity
Supplementary Methods S2.2. Multilocus Molecular Diagnosis
Supplementary Methods S2.3. Mosaicism and Parental Mosaicism
Supplementary Methods S2.4. Uniparental Disomy
Supplementary Methods S2.5. Mitochondrial DNA Heteroplasmy

Supplementary Figures

Supplementary Figure S1. Geographic Distribution of the Study Cohort
Supplementary Figure S2. Evolution of Genetic Testing Strategies, 2015–2024

Supplementary Tables

Supplementary Table S1. Patient-Level Genotypic and Diagnostic Characteristics of the Expanded Configuration Set
Supplementary Table S2. Clinical Phenotype–Gene Set Mapping and Principal Biological Functions Across Confirmed and Possibly Diagnostic Configurations
Supplementary Table S3. Temporal Sensitivity Analysis of Confirmed Diagnostic Configuration Contribution
Supplementary Table S4. Age at Onset Across Confirmed Diagnostic Configuration Categories
Supplementary Table S5. Sensitivity Analysis Using the Expanded Configuration Set
Supplementary Table S6. Expanded-Set Compound Heterozygous Configurations
Supplementary Table S7. Expanded-Set Multilocus Molecular Diagnoses
Supplementary Table S8. Expanded-Set Mosaic Findings and Candidate-Only Supplementary Records
Supplementary Table S9. Expanded-Set UPD-Related Configurations
Supplementary Table S10. Confirmed Diagnostic mtDNA Heteroplasmy Configurations

Supplementary Methods S1. Ethics Approval and Institutional Naming

The original ethics approval for this retrospective study was issued by the Institutional Review Board of the Capital Institute of Pediatrics (approval No. SHERLL2022064). The current institutional and clinical-service designation used in the manuscript reflects administrative updates during the study period; the approving ethics committee, study site, clinical testing database, and enrolled population were unchanged.

Supplementary Methods S2. Operational Classification of the Five Prespecified Configuration Categories

The main Methods section defines the patient-level diagnostic categories and overall configuration-level analytical framework. The operational criteria used to classify autosomal-recessive compound heterozygosity, multilocus molecular diagnoses, mosaicism, uniparental disomy (UPD), and mitochondrial DNA (mtDNA) heteroplasmy are detailed below. Classification was based on the clinical and laboratory evidence available for each child.

Supplementary Methods S2.1. Autosomal-Recessive Compound Heterozygosity

Compound heterozygous configurations comprised two different disease-associated alleles occurring in trans within the same autosomal-recessive disease gene. Eligible combinations included an SNV/indel plus another SNV/indel or an SNV/indel plus a copy-number variant (CNV) affecting the second allele. A configuration was retained only when the gene was associated with autosomal-recessive inheritance and the combined genotype was compatible with the child’s phenotype.

SNV/indel variants were detected by exome sequencing (ES) and interpreted according to ACMG/AMP standards. Allelic phase was assessed using trio ES, parental testing, segregation analysis, or other available family-based evidence. When both parental samples were available, trans inheritance was inferred when each parent carried one of the two alleles. When parental information was incomplete, the configuration was retained only when the available evidence supported a clinically plausible trans arrangement.

CNV second alleles were identified through ES read-depth-based CNV analysis, chromosomal microarray, SNP-array analysis, or targeted validation. CNV size, exon-level involvement, gene overlap, and compatibility with the expected loss-of-function or dosage mechanism were considered. Small CNVs below the resolution of conventional chromosomal microarray were retained when supported by ES read-depth data, orthogonal validation, or other clinically relevant evidence.

A configuration was classified as confirmed diagnostic when both alleles had diagnostic-level evidence, occurred in trans, and jointly explained the phenotype under an autosomal-recessive model. Possibly diagnostic status was assigned when the phenotype was compatible but one or both alleles lacked sufficient evidence for definitive classification, including P/LP plus VUS and VUS plus VUS configurations. Simple homozygous recessive diagnoses were excluded from this category.

Supplementary Methods S2.2. Multilocus Molecular Diagnosis

A multilocus molecular diagnosis comprised two or more independent molecular components in the same child. Each component could represent a monogenic dominant diagnosis, a biallelic recessive diagnosis, a CNV diagnosis, a UPD-related diagnosis, an mtDNA-related diagnosis, or another molecular finding that independently contributed to the phenotype.

Each molecular component was evaluated separately for variant pathogenicity, gene–disease validity, inheritance pattern, and phenotype compatibility. A multilocus configuration was classified as confirmed diagnostic only when at least two independent molecular components each had diagnostic-level evidence and explained distinct or partially overlapping components of the phenotype. Possibly diagnostic status was assigned when one component was confirmed and another was phenotype-compatible but lacked definitive evidence, or when two clinically relevant components both remained below the definitive evidence threshold.

Inheritance and de novo status of individual molecular components were assessed using trio ES or parental validation when available. Biallelic variants within the same autosomal-recessive gene, including an SNV/indel plus a CNV affecting the second allele, were classified as compound heterozygosity rather than as a multilocus molecular diagnosis.

Supplementary Methods S2.3. Mosaicism and Parental Mosaicism

The mosaicism category included both proband and parental mosaicism. Proband mosaicism was defined as a phenotype-relevant variant detected at a variant allele fraction (VAF) below that expected for constitutional heterozygosity or showing a tissue-restricted distribution. Proband/de novo mosaicism included postzygotic variants in the child and de novo findings with a mosaic-range VAF.

Parental mosaicism was defined by molecular evidence of reduced or atypical allelic representation relative to constitutional heterozygosity, tissue-specific variation, abnormal sequencing peak patterns, or other laboratory evidence supporting somatic, germline, or gonosomal mosaicism. Parental findings were classified as maternal or paternal according to the parent carrying the mosaic variant.

Mosaicism was detected or suspected through ES, targeted next-generation sequencing panels, Sanger sequencing peak imbalance, high-depth targeted sequencing, or tissue-specific testing. Peripheral blood was the principal tested tissue. Non-blood or affected tissues were reviewed when available, particularly for suspected tissue-restricted postzygotic events. Sequencing depth, VAF, tissue source, inheritance pattern, and validation method were recorded when available.

A mosaic finding was classified as confirmed diagnostic when a P/LP variant was compatible with the phenotype and the mosaic state was adequately supported by VAF, tissue distribution, parental analysis, or orthogonal validation. Possibly diagnostic status was assigned when the phenotype and mosaic pattern were compatible but variant classification or confirmatory evidence remained insufficient. Candidate-only findings were retained for supplementary reporting when mosaicism was suspected but the available evidence did not support inclusion in the expanded configuration set.

Variants with VAFs approaching the constitutional heterozygous range were considered mosaic only when supported by additional molecular, segregation, or tissue-specific evidence.

Supplementary Methods S2.4. Uniparental Disomy

The UPD category included isodisomy, heterodisomy, and mixed UPD events that caused an imprinting disorder or contributed to diagnosis by unmasking a recessive pathogenic allele. Interpretation incorporated parental origin, long contiguous stretches of homozygosity, relevant imprinting regions, recessive alleles, and phenotype compatibility.

UPD was detected or supported through chromosomal microarray, SNP-array analysis, trio-based genotype analysis, long contiguous stretches of homozygosity, parental-origin analysis, or category-appropriate confirmatory testing. Parental SNP-array or other parental-origin analyses were reviewed when clinically performed. Methylation results were also considered when available for imprinting-related disorders.

A UPD configuration was classified as confirmed diagnostic when parental origin and the affected chromosome or region were consistent with a recognized imprinting disorder or when isodisomy unmasked a pathogenic recessive allele in a phenotype-compatible gene. Possibly diagnostic status was assigned when the event was phenotype-compatible but parental origin, methylation status, or category-appropriate confirmation was incomplete.

UPD-like homozygosity patterns without phenotype compatibility, a relevant imprinting or recessive mechanism, or adequate support for parental origin were excluded from the expanded configuration set. X-chromosome isodisomy was not interpreted as a classical autosomal imprinting-related UPD event; the retained X-chromosome isodisomy finding was therefore reported with a configuration-specific annotation.

Supplementary Methods S2.5. Mitochondrial DNA Heteroplasmy

The mtDNA heteroplasmy category included pathogenic or likely pathogenic mtDNA variants for which quantitative variant-level information was available. For each finding, the mtDNA variant, heteroplasmy level, tested tissue, maternal testing result, inheritance pattern, and clinical phenotype were recorded.

Variants were detected by full-length mitochondrial genome next-generation sequencing or other clinically performed quantitative mtDNA testing. Interpretation considered variant pathogenicity, phenotype compatibility, heteroplasmy level, tissue source, and maternal inheritance. Maternal inheritance was assessed using available maternal testing or documented maternal-line segregation.

An mtDNA configuration was classified as confirmed diagnostic when the variant was pathogenic or likely pathogenic, the measured variant level and tissue source were clinically interpretable, and the phenotype was compatible with the associated mitochondrial disease spectrum. Findings not detected in available maternal samples were classified according to the available clinical and laboratory evidence.

When multiple tissues were tested, differences in heteroplasmy levels were documented. Blood–urine heteroplasmy differences were summarized descriptively when paired measurements were available.

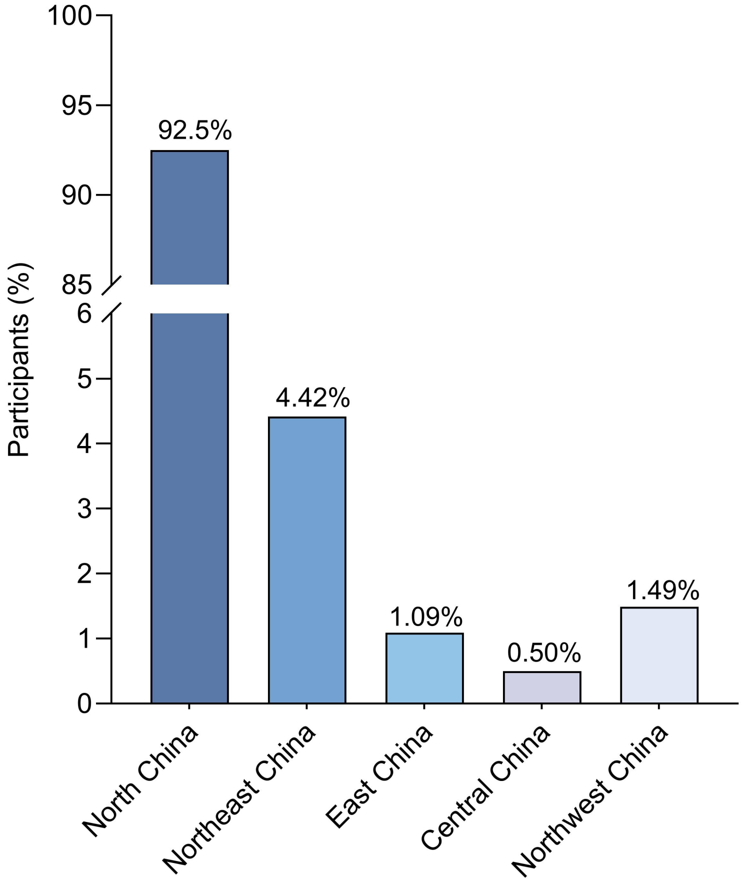

Supplementary Figure S1. Geographic Distribution of the Study Cohort.

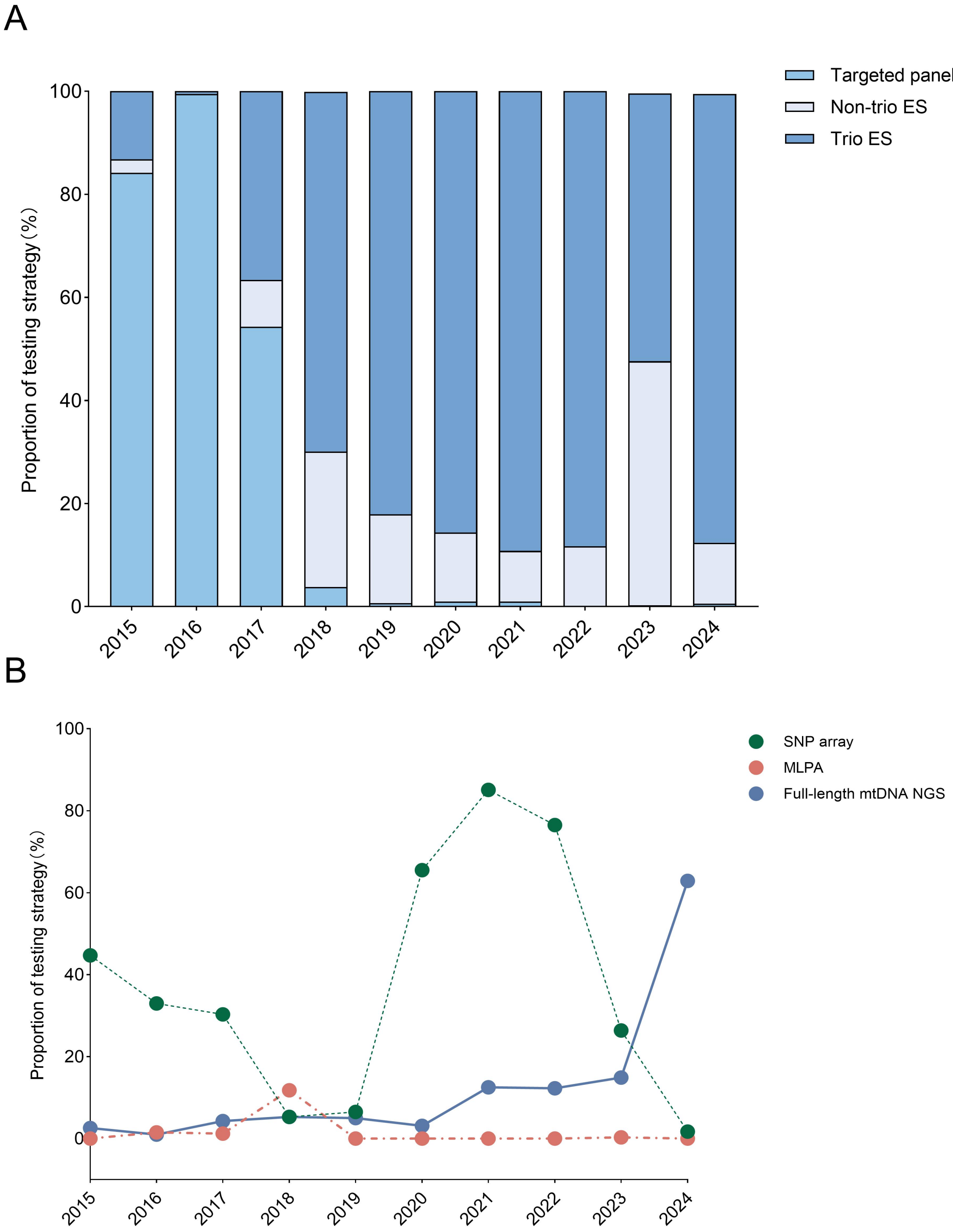

Supplementary Figure S2. Evolution of Genetic Testing Strategies, 2015–2024. (A) Annual distribution of the main nuclear genetic testing strategies, including targeted-panel sequencing, non-trio exome sequencing (ES), and trio ES. These three categories were mutually exclusive. (B) Annual proportions of children undergoing SNP-array analysis, multiplex ligation-dependent probe amplification (MLPA), or full-length mtDNA NGS. These testing modalities were not mutually exclusive with the main nuclear genetic testing strategies or with one another. Percentages were calculated using the total number of children tested in each calendar year as the denominator. Abbreviations: ES, exome sequencing, MLPA, multiplex ligation-dependent probe amplification, mtDNA, mitochondrial DNA, NGS, next-generation sequencing.

Supplementary Table S1. Patient-Level Genotypic and Diagnostic Characteristics of the Expanded Configuration Set

Case ID	Configuration category	Diagnostic category	Sex	Age at onset (y)	Phenotype	Gene	Variant information	Testing
CH001	Compound heterozygosity	Confirmed diagnostic	Male	0.8	Epilepsy; GDD/ID; Abnormal muscle tone	*TSEN2*	c.926A>G; ex.7del	ES （trio） + SNP array
CH002	Compound heterozygosity	Confirmed diagnostic	Female	8.3	GDD/ID	*MTHFR*	[c.136C>T; c.698C>G]; ex.1-3del	Targeted panel + SNP array
CH003	Compound heterozygosity	Confirmed diagnostic	Male	1.1	GDD/ID; Abnormal muscle tone	*SMN1*	c.399_402del; ex.7del	ES （trio） + SNP array
CH004	Compound heterozygosity	Confirmed diagnostic	Male	15.1	Epilepsy	*ATP7B*	c.2333G>T; ex.17del	ES （trio） + SNP array
CH005	Compound heterozygosity	Confirmed diagnostic	Male	0.3	Epilepsy; GDD/ID	*POLG*	c.3218C>T; ex.1-23del	ES （trio） + SNP array
CH006	Compound heterozygosity	Confirmed diagnostic	Male	0.1	Epilepsy	*ALDH7A1*	c.1008+1G>A; ex.6del	ES （trio） + SNP array
CH007	Compound heterozygosity	Confirmed diagnostic	Female	2	GDD/ID; Facial dysmorphism	*WDR62*	c.1312C>T; ex.10-20dup	ES （trio） + SNP array
CH008	Compound heterozygosity	Confirmed diagnostic	Male	0.3	GDD/ID	*ETFDH*	c.1531G>C; ex.1-6del	ES （trio） + SNP array
CH009	Compound heterozygosity	Confirmed diagnostic	Female	4.4	GDD/ID	*ERCC8*	c.394_398del; ex.4del	Targeted panel + SNP array
CH010	Compound heterozygosity	Confirmed diagnostic	Male	3.3	GDD/ID; Facial dysmorphism; Microcephaly	*ERCC8*	c.173+5del; ex.4del	ES （trio） + SNP array

CH011	Compound heterozygosity	Confirmed diagnostic	Female	0.4	GDD/ID	*DDC*	c.557A>G; ex.11-12del	ES（trio）+ SNP array
CH012	Compound heterozygosity	Confirmed diagnostic	Male	2.4	GDD/ID; Abnormal muscle tone	*FARS2*	c.1219A>G; ex.2-3del	ES（trio）+ SNP array
CH013	Compound heterozygosity	Confirmed diagnostic	Female	0.7	Epilepsy	*DOCK6*	c.3190_3191delCT; c.873+1G>A	Targeted panel
CH014	Compound heterozygosity	Confirmed diagnostic	Male	3.1	GDD/ID; Abnormal muscle tone	*ARSA*	c.1175G>A; c.893G>T	ES（trio）
CH015	Compound heterozygosity	Confirmed diagnostic	Male	0.2	Epilepsy	*ACTL6B*	c.11delG; c.937-2A>G	ES（trio）
CH016	Compound heterozygosity	Confirmed diagnostic	Female	3	Epilepsy	*ALDH7A1*	c.1093+2T>C; c.1547A>G	ES（trio）
CH017	Compound heterozygosity	Confirmed diagnostic	Male	0.17	Epilepsy	*PAH*	c.721C>T; c.971T>A	Targeted panel
CH018	Compound heterozygosity	Confirmed diagnostic	Female	1.1	GDD/ID; Abnormal muscle tone	*GAA*	c.1447G>A; c.2662G>T	Targeted panel
CH019	Compound heterozygosity	Confirmed diagnostic	Female	10.2	Epilepsy	*CYP27A1*	c.1415G>C; c.379C>T	ES（trio）
CH020	Compound heterozygosity	Confirmed diagnostic	Female	0.02	Epilepsy	*TYR*	c.832C>T; c.929dupC	Targeted panel
CH021	Compound heterozygosity	Confirmed diagnostic	Male	1.2	GDD/ID	*TH*	c.457C>T; c.698G>A	ES（trio）
CH022	Compound heterozygosity	Confirmed diagnostic	Female	11.8	GDD/ID	*PANK2*	c.1069C>T; c.595C>T	ES（trio）
CH023	Compound heterozygosity	Confirmed diagnostic	Male	0.8	GDD/ID	*ECHS1*	c.229G>A; c.5C>T	ES（trio）

CH024	Compound heterozygosity	Confirmed diagnostic	Male	0.14	Epilepsy	*PCCB*	c.1087T>C; c.369_372delTCAG	Targeted panel
CH025	Compound heterozygosity	Confirmed diagnostic	Male	3	Epilepsy	*PAH*	c.1068C>A; c.971T>A	ES（trio）
CH026	Compound heterozygosity	Confirmed diagnostic	Male	1.1	GDD/ID; Abnormal muscle tone	*TH*	c.457C>T; c.698G>A	ES（trio）
CH027	Compound heterozygosity	Confirmed diagnostic	Female	3.05	Epilepsy	*POMT2*	c.1261C>T; c.1762C>T	ES（trio）
CH028	Compound heterozygosity	Confirmed diagnostic	Male	3.3	GDD/ID	*FA2H*	c.950_953del; c.968C>T	ES（trio）
CH029	Compound heterozygosity	Confirmed diagnostic	Male	12	Epilepsy	*WISP3*	c.589+2T>C; c.667T>G	ES（trio）
CH030	Compound heterozygosity	Confirmed diagnostic	Male	1.8	GDD/ID; Abnormal muscle tone	*ARSA*	c.185_186dup; c.917C>T	ES（trio）
CH031	Compound heterozygosity	Confirmed diagnostic	Male	0.9	Epilepsy; GDD/ID	*HEXA*	c.1436del; c.1444G>A	ES（trio）
CH032	Compound heterozygosity	Confirmed diagnostic	Female	17	GDD/ID	*L2HGDH*	c.407del; c.800_801del	Targeted panel
CH033	Compound heterozygosity	Confirmed diagnostic	Male	0.4	Epilepsy	*RARS2*	c.1A>C; c.772C>T	ES（duo）
CH034	Compound heterozygosity	Confirmed diagnostic	Male	0.4	GDD/ID	*AHI1*	c.1303C>T; c.1799_1802delAACA	ES（trio）
CH035	Compound heterozygosity	Confirmed diagnostic	Female	0.7	Epilepsy	*ACADS*	c.1031A>G; c.989G>A	ES（trio）
CH036	Compound heterozygosity	Confirmed diagnostic	Male	0.73	Epilepsy	*GCDH*	c.1159G>C; c.416C>G	ES（trio）

CH037	Compound heterozygosity	Confirmed diagnostic	Female	0.6	GDD/ID	*VPS13B*	c.11695_11698del; c.5230C>T	ES（trio）
CH038	Compound heterozygosity	Confirmed diagnostic	Female	0.3	Epilepsy	*ST3GAL5*	c.1000delC; c.601G>A	ES（trio）
CH039	Compound heterozygosity	Confirmed diagnostic	Female	0.7	Epilepsy; GDD/ID	*MTHFR*	c.1003C>T; c.1632+2T>G	Targeted panel
CH040	Compound heterozygosity	Confirmed diagnostic	Male	0.2	Epilepsy	*TH*	c.698G>A; c.943G>A	ES（trio）
CH041	Compound heterozygosity	Confirmed diagnostic	Male	2	GDD/ID	*ATM*	c.4396C>T; c.6572+1G>T	Targeted panel
CH042	Compound heterozygosity	Confirmed diagnostic	Female	1.57	Epilepsy	*SLC22A5*	c.497+1G>T; c.839C>A	ES（trio）
CH043	Compound heterozygosity	Confirmed diagnostic	Male	6	Epilepsy; GDD/ID	*PLA2G6*	c.1634A>G; c.238G>A	ES（trio）
CH044	Compound heterozygosity	Confirmed diagnostic	Male	8.41	ASD; GDD/ID	*ETFDH*	c.389A>T; c.770A>G	ES（trio）
CH045	Compound heterozygosity	Confirmed diagnostic	Male	2.1	Epilepsy; Abnormal muscle tone	*HIBCH*	c.1027C>G; c.439-2A>G	ES（trio）
CH046	Compound heterozygosity	Confirmed diagnostic	Female	1.64	Epilepsy	*TYR*	c.155G>T; c.455C>A	ES（trio）
CH047	Compound heterozygosity	Confirmed diagnostic	Female	5.2	GDD/ID	*ABCC2*	c.1326G>A; c.2443C>T	ES（trio）
CH048	Compound heterozygosity	Confirmed diagnostic	Female	5.9	GDD/ID	*CYP2U1*	c.1380_1416del; c.471del	ES（duo）
CH049	Compound heterozygosity	Confirmed diagnostic	Male	3.3	GDD/ID	*BCS1L*	c.245C>A; c.548G>A	Targeted panel

CH050	Compound heterozygosity	Confirmed diagnostic	Female	0.33	Epilepsy	*PAH*	c.1054G>C; c.728G>A	ES（trio）
CH051	Compound heterozygosity	Confirmed diagnostic	Male	11.3	GDD/ID; ASD	*NALCN*	c.2340delA; c.5173_5176dupACTG	ES（trio）
CH052	Compound heterozygosity	Confirmed diagnostic	Male	3.33	Epilepsy	*FKRP*	c.545A>G; c.941C>T	Targeted panel
CH053	Compound heterozygosity	Confirmed diagnostic	Male	3	GDD/ID; Abnormal muscle tone	*TH*	c.1282G>A; c.739G>A	Targeted panel
CH054	Compound heterozygosity	Confirmed diagnostic	Female	1	Epilepsy	*TSFM*	c.161G>A; c.971_972delAG	ES（trio）
CH055	Compound heterozygosity	Confirmed diagnostic	Male	3.85	Epilepsy	*UNC13D*	c.118-308C>T; c.1241G>T	ES（trio）
CH056	Compound heterozygosity	Confirmed diagnostic	Female	0.09	Epilepsy	*PAH*	c.1068C>A; c.1238G>C	Targeted panel
CH057	Compound heterozygosity	Confirmed diagnostic	Female	0.3	Epilepsy	*WWOX*	c.132G>A; c.49G>A	ES（trio）
CH058	Compound heterozygosity	Confirmed diagnostic	Female	1.6	GDD/ID	*PMM2*	c.430T>C; c.531G>C	ES（trio）
CH059	Compound heterozygosity	Confirmed diagnostic	Male	1.25	Epilepsy	*GALC*	c.467_468dupGG; c.469_470delC	ES（trio）
CH060	Compound heterozygosity	Confirmed diagnostic	Male	1.9	Epilepsy; GDD/ID	*PIGN*	c.1172+1C>G; c.1434+5C>T	Targeted panel
CH061	Compound heterozygosity	Confirmed diagnostic	Female	5.48	Epilepsy	*PRF1*	c.1349C>T; c.65delC	ES（trio）
CH062	Compound heterozygosi	Confirmed diagnostic	Male	1.1	Epilepsy	*MUT*	c.1718T>C; c.323G>A	ES（trio）

	ty							
CH063	Compound heterozygosity	Confirmed diagnostic	Male	5.4	GDD/ID	*PAH*	c.116_118del; c.442-1G>A	ES (trio)
CH064	Compound heterozygosity	Confirmed diagnostic	Male	1.92	Abnormal muscle tone	*TH*	c.524T>C; c.698G>A	ES (trio)
CH065	Compound heterozygosity	Confirmed diagnostic	Female	Unknown	Epilepsy	*FA2H*	c.613+2T>C; c.806G>T	ES (trio)
CH066	Compound heterozygosity	Possibly diagnostic	Male	2.9	Epilepsy	*SLC12A3*	c.1670-7G>A; c.1925G>A	ES (trio)
CH067	Compound heterozygosity	Confirmed diagnostic	Female	0.3	Epilepsy	*MMACHC*	c.567dup; c.609G>A	ES (trio)
CH068	Compound heterozygosity	Confirmed diagnostic	Male	6	GDD/ID	*MMACHC*	c.365A>T; c.658_660del	ES (trio)
CH069	Compound heterozygosity	Confirmed diagnostic	Male	1	Epilepsy	*MMACHC*	c.609G>A; c.658_660del	ES (trio)
CH070	Compound heterozygosity	Confirmed diagnostic	Male	5.83	Epilepsy	*MMACHC*	c.394C>T; c.658_660del	ES (trio)
CH071	Compound heterozygosity	Confirmed diagnostic	Female	0.2	Epilepsy	*MMACHC*	c.394C>T; c.626dup	ES (trio)
CH072	Compound heterozygosity	Confirmed diagnostic	Male	0.2	Epilepsy	*MMACHC*	c.567dup; c.609G>A	ES (trio)
CH073	Compound heterozygosity	Confirmed diagnostic	Female	0.4	Epilepsy; Abnormal muscle tone	*MMACHC*	c.609G>A; c.658_660del	ES (trio)
CH074	Compound heterozygosity	Confirmed diagnostic	Female	16	Movement/ motor or gait disorder	*MMACHC*	c.575T>C; c.1A>G	ES (trio)

CH075	Compound heterozygosity	Confirmed diagnostic	Male	0.7	GDD/ID	*MMACHC*	c.1A>G; c.271A>T	ES（trio）
CH076	Compound heterozygosity	Confirmed diagnostic	Male	9	Epilepsy	*MMACHC*	c.277-3_303del; c.482G>A	ES（trio）
CH077	Compound heterozygosity	Confirmed diagnostic	Male	0.4	GDD/ID; Abnormal muscle tone	*MMACHC*	c.626_627delTG; c.80A>G	ES（trio）
CH078	Compound heterozygosity	Confirmed diagnostic	Female	6.67	Epilepsy	*MMACHC*	c.445_446insA; c.482G>A	ES（trio）
CH079	Compound heterozygosity	Confirmed diagnostic	Male	0.4	GDD/ID	*MMACHC*	c.481C>T; c.658_660del	ES（trio）
CH080	Compound heterozygosity	Confirmed diagnostic	Male	2.58	Epilepsy	*MMACHC*	c.484G>A; c.567dup	Targeted panel
CH081	Compound heterozygosity	Confirmed diagnostic	Female	15.75	Movement/motor or gait disorder	*MMACHC*	c.1A>G; c.609G>A	ES（trio）
CH082	Compound heterozygosity	Confirmed diagnostic	Female	0.4	GDD/ID; Microcephaly	*MMACHC*	c.658_660del; c.666C>A	ES（trio）
CH083	Compound heterozygosity	Confirmed diagnostic	Male	0.33	Epilepsy	*MMACHC*	c.609G>A; c.658_660del	ES（trio）
CH084	Compound heterozygosity	Confirmed diagnostic	Male	0.8	GDD/ID	*MMACHC*	c.567dup; c.609G>A	Targeted panel
CH085	Compound heterozygosity	Confirmed diagnostic	Male	0.21	Epilepsy	*MMACHC*	c.445_446del; c.609G>A	ES（trio）
CH086	Compound heterozygosity	Confirmed diagnostic	Male	0.26	Epilepsy	*MMACHC*	c.394C>T; c.567dup	ES（trio）
CH087	Compound heterozygosity	Confirmed diagnostic	Female	0.3	Epilepsy	*MMACHC*	c.315C>G; c.609G>A	ES（trio）

CH088	Compound heterozygosity	Confirmed diagnostic	Male	11	Movement/ motor or gait disorder	*MMACHC*	c.482G>A; c.656_658del	ES（trio）
CH089	Compound heterozygosity	Confirmed diagnostic	Male	10	Epilepsy	*MMACHC*	c.482G>A; c.567dup	ES（trio）
CH090	Compound heterozygosity	Confirmed diagnostic	Male	9	GDD/ID	*MMACHC*	c.365A>T; c.609G>A	ES（trio）
CH091	Compound heterozygosity	Confirmed diagnostic	Male	0.09	GDD/ID	*MMACHC*	c.481C>T; c.609G>A	ES（trio）
CH092	Compound heterozygosity	Confirmed diagnostic	Male	6	Epilepsy	*MMACHC*	c.482G>A; c.609G>A	ES（trio）
CH093	Compound heterozygosity	Confirmed diagnostic	Male	16	Movement/ motor or gait disorder	*MMACHC*	c.565C>G; c.609G>A	ES（trio）
CH094	Compound heterozygosity	Confirmed diagnostic	Male	0.01	GDD/ID; Abnormal muscle tone	*MMACHC*	c.80A>G; c.609G>A	ES（trio）
CH095	Compound heterozygosity	Confirmed diagnostic	Male	Unknown	Epilepsy	*MMACHC*	c.609G>A; c.271dupA	ES（trio）
CH096	Compound heterozygosity	Confirmed diagnostic	Male	5	Movement/ motor or gait disorder	*MMACHC*	c.277-3_303del; c.470G>C	ES（trio）
CH097	Compound heterozygosity	Confirmed diagnostic	Female	0.58	Abnormal muscle tone	*MMACHC*	c.365A>T; c.609G>A	ES（trio）
CH098	Compound heterozygosity	Confirmed diagnostic	Male	3.8	Epilepsy	*MMACHC*	c.1A>G; c.617G>A	ES（trio）
CH099	Compound heterozygosity	Confirmed diagnostic	Male	18	Movement/ motor or gait disorder	*MMACHC*	c.482G>A; c.445_446insA	ES（trio）
CH100	Compound heterozygosity	Confirmed diagnostic	Female	18	GDD/ID	*MMACHC*	c.1A>G; c.445_446delTG	ES（trio）

CH101	Compound heterozygosity	Confirmed diagnostic	Male	12.9	GDD/ID; ASD	*MMACHC*	c.354G>C; c.80A>G	ES（trio）
CH102	Compound heterozygosity	Confirmed diagnostic	Male	0.3	GDD/ID	*MMACHC*	c.609G>A; c.80A>G	ES（trio）
CH103	Compound heterozygosity	Confirmed diagnostic	Male	0.3	Epilepsy	*MMACHC*	c.609G>A; c.80A>G	ES（trio）
CH104	Compound heterozygosity	Confirmed diagnostic	Male	0.5	GDD/ID; ASD; Abnormal muscle tone	*MMACHC*	c.482G>A; c.658_660del	ES（trio）
ML001	Multilocus molecular diagnosis	Confirmed diagnostic	Male	0.8	GDD/ID	*SOX5; NIPA1*	SOX5: c.1477C>T; NIPA1: chr15:22,833,374–23,406,431 deletion	ES（proband）
ML002	Multilocus molecular diagnosis	Possibly diagnostic	Female	0.5	GDD/ID	*ADA2; IL17RA*	NA	ES（trio）+ SNP array
ML003	Multilocus molecular diagnosis	Possibly diagnostic	Male	7.4	GDD/ID	*ANK3; IL1RAPL1*	ANK3: c.2261C>T;c.5000C>A; IL1RAPL1:c.1195G>A	ES（trio）+ SNP array
ML004	Multilocus molecular diagnosis	Possibly diagnostic	Male	0.5	Epilepsy	*CIT; STK11*	CIT: c.190C>T; STK11: chr19:871,106–1,329,639 deletion	ES（trio）+ SNP array
ML005	Multilocus molecular diagnosis	Confirmed diagnostic	Female	1	GDD/ID	*SLC6A1; TCF4*	SLC6A1: c.1695+2T>A; TCF4: exon 6 deletion	ES（trio）+ SNP array
ML006	Multilocus molecular diagnosis	Confirmed diagnostic	Male	0.7	Epilepsy, abnormal muscle tone	*PRRT2; PTEN*	PRRT2: c.649dup; PTEN:	ES（trio）+ SNP array

							c.518G>A	
ML007	Multilocus molecular diagnosis	Possibly diagnostic	Female	9.8	GDD/ID	*FAM20C; PDGFA;*	NA	ES（trio）
ML008	Multilocus molecular diagnosis	Possibly diagnostic	Male	4	GDD/ID	*ANKH; DNAH5*	NA	ES（trio）
ML009	Multilocus molecular diagnosis	Confirmed diagnostic	Male	0.5	Epilepsy	*CDH2; PHACTR1*	CDH2: c.949del; PHACTR1: c.1495_1497del	ES（trio）
ML010	Multilocus molecular diagnosis	Possibly diagnostic	Male	9.8	Epilepsy	*KCNT1; PRRT2*	KCNT1: c.1249C>T; PRRT2: c.883C>T	Targeted panel
ML011	Multilocus molecular diagnosis	Confirmed diagnostic	Male	0.2	Epilepsy	*DHX30; SCN1A*	DHX30: c.2266C>T; SCN1A: chr2:165,754,688–168,986,136×3	ES（trio）+ SNP array
ML012	Multilocus molecular diagnosis	Confirmed diagnostic	Female	2.5	Epilepsy, abnormal muscle tone	*KCNQ2; ZMYND11*	KCNQ2: c.619C>T; ZMYND11: c.868_877dup	ES（trio）
ML013	Multilocus molecular diagnosis	Confirmed diagnostic	Female	2.6	Epilepsy, GDD/ID	*BAG3; CSNK2B*	BAG3: c.626C>T; CSNK2B: c.443C>A	ES（trio）
ML014	Multilocus molecular diagnosis	Possibly diagnostic	Male	7.5	Epilepsy, GDD/ID	*NDP; TRRAP*	NDP: c.134_135del; TRRAP: c.7330T>G	ES（trio）

ML015	Multilocus molecular diagnosis	Possibly diagnostic	Male	1	Microcephaly, abnormal muscle tone, GDD/ID	*AKT3; ZBTB18*	AKT3: NA; ZBTB18: c.583C>T	ES（trio）+ SNP array
ML016	Multilocus molecular diagnosis	Confirmed diagnostic	Male	5	ASD, facial dysmorphism, microcephaly, GDD/ID	*DMD; GDI1*	DMD: exon 51 deletion; GDI1: c.1159delT	ES（trio）+ SNP array
ML017	Multilocus molecular diagnosis	Confirmed diagnostic	Female	1.5	Epilepsy	*SCN1A; HNF1B*	SCN1A: c.4021_4022delTT; HNF1B-region deletion: chr17:34,817,693–36,249,430×1	ES（trio）+ SNP array
ML018	Multilocus molecular diagnosis	Possibly diagnostic	Female	5.5	Epilepsy, GDD/ID	*IQSEC2; KCNT2*	IQSEC2: c.2507C>T; KCNT2: c.3031C>T	ES（trio）
ML019	Multilocus molecular diagnosis	Confirmed diagnostic	Female	0.6	GDD/ID	*RGS10; TIAL1*	NA	ES（trio）+ SNP array
ML020	Multilocus molecular diagnosis	Confirmed diagnostic	Female	2.2	Abnormal muscle tone, GDD/ID	*ARID1B; SHANK3*	ARID1B: c.1041_1071del; SHANK3-region deletion: chr22:48,871,083–51,213,613×1	ES（trio）+ SNP array
ML021	Multilocus molecular diagnosis	Possibly diagnostic	Male	0.5	Epilepsy	*SCN1A; SCN8A*	SCN1A: c.664C>T; SCN8A: c.4711C>T	ES（trio）

ML022	Multilocus molecular diagnosis	Confirmed diagnostic	Male	9	GDD/ID	*MMACHC; POLR2A*	MMACHC: c.658_660del; POLR2A: c.3700G>A	ES（trio）
ML023	Multilocus molecular diagnosis	Possibly diagnostic	Female	2.6	Epilepsy	*PRRT2; AATF*	PRRT2: c.649insC; AATF:NA	ES（trio）+ SNP array
ML024	Multilocus molecular diagnosis	Possibly diagnostic	Female	4	ASD, abnormal muscle tone, GDD/ID	*GLB1; SGSH*	GLB1: c.1833G>A; SGSH: c.571G>A	ES（trio）
ML025	Multilocus molecular diagnosis	Confirmed diagnostic	Female	3.6	Epilepsy, GDD/ID	*CARKD; CARS2*	NA	ES（trio）
ML026	Multilocus molecular diagnosis	Possibly diagnostic	Male	2.8	Epilepsy	*SCN8A; SCN9A*	SCN8A: c.4711C>T; SCN9A: c.1444A>C	ES（trio）
ML027	Multilocus molecular diagnosis	Confirmed diagnostic	Male	7.3	Epilepsy, GDD/ID	*PPP2R1A; SCN1A*	PPP2R1A: c.442T>C; SCN1A: c.1336C>T	ES（trio）
ML028	Multilocus molecular diagnosis	Possibly diagnostic	Male	0.9	GDD/ID	*GAMT; SETD5*	GAMT: c.265_267del; SETD5: c.1312G>A	ES（trio）
ML029	Multilocus molecular diagnosis	Confirmed diagnostic	Male	1.4	Abnormal muscle tone, facial dysmorphism, GDD/ID	*HDAC4; AGAP1*	HDAC4: chr2:66,415,349–100,247,142; AGAP1: NA	Targeted panel + SNP array
ML030	Multilocus molecular diagnosis	Possibly diagnostic	Female	0.3	Epilepsy, GDD/ID	*ITGB6; PKP4*	NA	ES（trio）+ SNP array
ML031	Multilocus molecular diagnosis	Possibly diagnostic	Male	3.5	Epilepsy, GDD/ID	*MFSD8; SCN8A*	MFSD8: c.217dupA; SCN8A: c.5720G>A	ES（trio）
ML032	Multilocus molecular diagnosis	Confirmed diagnostic	Male	3.4	Abnormal muscle tone, GDD/ID	*G6PD; GNAO1*	G6PD: c.1376G>T; GNAO1:	ES（trio）

							c.607G>A	
ML033	Multilocus molecular diagnosis	Possibly diagnostic	Male	3.8	Epilepsy	*NPRL2; SCN1A*	NPRL2: c.475G>A; SCN1A: c.5582G>A	ES （proband）
ML034	Multilocus molecular diagnosis	Confirmed diagnostic	Male	0.3	Epilepsy	*CBLN4; MC3R*	NA	ES （trio） + SNP array
ML035	Multilocus molecular diagnosis	Confirmed diagnostic	Male	1.8	Epilepsy, GDD/ID, facial dysmorphism	*STXBP1; NF1*	STXBP1: c.1193A>G; NF1: chr17:29,111,368–30,208,636 ×1	ES （trio） + SNP array
ML036	Multilocus molecular diagnosis	Possibly diagnostic	Female	1	Epilepsy, abnormal muscle tone, GDD/ID	*COL4A2; SLC13A5*	COL4A2: c.481C>A; SLC13A5: c.410T>C	ES （trio）
ML037	Multilocus molecular diagnosis	Possibly diagnostic	Male	8	GDD/ID	*GABRG2; TTBK2*	GABRG2: c.1119delG; TTBK2: c.1589G>A	ES （proband）
ML038	Multilocus molecular diagnosis	Possibly diagnostic	Male	14.4	GDD/ID, facial dysmorphism	*KDM5C; TUBB4A*	KDM5C: c.1127G>C; TUBB4A: c.1091C>A	ES （proband）
ML039	Multilocus molecular diagnosis	Confirmed diagnostic	Male	2.7	GDD/ID	*SHANK3; UBE3A*	SHANK3: c.2499delG; UBE3A: chr15:25,650,587–26,108,369 ×3	ES （duo）
ML040	Multilocus molecular diagnosis	Possibly diagnostic	Female	1.9	Facial dysmorphism, GDD/ID	*DDX6; ACKR3*	DDX6: c.833C>G; ACKR3: chr2:235,961,972–243,178,150	ES （trio） + SNP array

							×1	
ML041	Multilocus molecular diagnosis	Confirmed diagnostic	Female	16.8	Epilepsy	*ATP7B; CHD2*	ATP7B: c.2333G>T; CHD2: c.1373G>A	ES（trio）
ML042	Multilocus molecular diagnosis	Confirmed diagnostic	Male	1.5	GDD/ID	*CACNA1A; SYNGAP1*	CACNA1A: c.5455C>T; SYNGAP1: c.3233_3236 delTCAG	ES（trio）
ML043	Multilocus molecular diagnosis	Possibly diagnostic	Female	6.8	GDD/ID	*MAGEL2; SNX14*	MAGEL2: c.290del; SNX14: c.2269-6T>G and exons 7–26 deletion	ES（trio）+ SNP array
ML044	Multilocus molecular diagnosis	Confirmed diagnostic	Male	2	ASD, GDD/ID	*CHD2; SCN2A; STS*	CHD2: c.1373G>A; SCN2A: c.605+1G>A; STS-region Xp22.31 deletion	ES（trio）+ SNP array
ML045	Multilocus molecular diagnosis	Confirmed diagnostic	Female	0.4	Epilepsy, GDD/ID	*SCN8A; MCPH1*	SCN8A: c.2668G>C; MCPH1: chr8:176,273–7,274,004 ×1	ES（trio）+ SNP array
ML046	Multilocus molecular diagnosis	Confirmed diagnostic	Male	0.6	Epilepsy	*FOXP1; PRRT2*	FOXP1: c.1172delT; PRRT2: chr16:29,595,483–30,327,917 ×1	ES（trio）+ SNP array

ML047	Multilocus molecular diagnosis	Possibly diagnostic	Female	0.1	Epilepsy	*LYST; MOGS*	LYST: c.8624G>A; MOGS: c.1061G>A	ES（proband）
ML048	Multilocus molecular diagnosis	Possibly diagnostic	Male	9.7	GDD/ID	*NF1; DMD*	NF1: c.4537C>T; DMD: exon 1 duplication	ES（proband）
MO001	Mosaicism	Confirmed diagnostic	Male	2.2	Epilepsy, microcephaly, GDD/ID, facial dysmorphism	*KMT2D*	c.10134dup	Targeted panel + SNP array
MO002	Mosaicism	Confirmed diagnostic	Male	1.5	Epilepsy, microcephaly, GDD/ID	*CASK*	c.2338G>T	Targeted panel + SNP array
MO003	Mosaicism	Possibly diagnostic	Female	4.64	Epilepsy	*FLNA*	c.2378C>A	ES（trio）+ SNP array
MO004	Mosaicism	Confirmed diagnostic	Female	4.05	Epilepsy, GDD/ID	*SMC1A*	c.3145C>T	ES（trio）+ SNP array
MO005	Mosaicism	Confirmed diagnostic	Male	0.6	Epilepsy	*CDKL5*	c.587C>T	ES（trio）+ SNP array
MO006	Mosaicism	Confirmed diagnostic	Male	0.59	Epilepsy, GDD/ID	*SLC35A2*	c.221C>T	ES（trio）+ SNP array
MO007	Mosaicism	Confirmed diagnostic	Female	5	Epilepsy	*ACTB*	c.439C>T	ES（trio）
MO008	Mosaicism	Confirmed diagnostic	Female	0.15	Epilepsy	*KRAS*	c.35G>A	ES（trio）+ SNP array
MO009	Mosaicism	Confirmed diagnostic	Female	0.81	Epilepsy	*PCDH19*	c.344_345delTA	ES（trio）+ SNP array
MO010	Mosaicism	Confirmed diagnostic	Female	4.7	GDD/ID	*STXBP1*	c.1301dupC	ES（trio）+ SNP array

MO011	Mosaicism	Confirmed diagnostic	Female	0.5	Epilepsy	*SCN1A*	c.664C>T	ES（trio）
MO012	Mosaicism	Possibly diagnostic	Female	2	Epilepsy, GDD/ID, abnormal muscle tone	*CDKL5*	c.515T>G	Targeted panel
MO013	Mosaicism	Confirmed diagnostic	Female	0.96	Epilepsy, GDD/ID	*ARX*	c.665_666dup	ES（trio） + SNP array
MO014	Mosaicism	Confirmed diagnostic	Male	9.75	Epilepsy, abnormal muscle tone, GDD/ID	*SLC2A1*	c.971C>T	ES（trio） + SNP array
MO015	Mosaicism	Confirmed diagnostic	Male	1	GDD/ID, abnormal muscle tone, microcephaly	*IQSEC2*	c.2507C>T	ES（trio） + SNP array
MO016	Mosaicism	Confirmed diagnostic	Male	3	GDD/ID	*PDHA1*	c.236A>T	ES（trio）
MO017	Mosaicism	Confirmed diagnostic	Female	0.18	Epilepsy, GDD/ID	*SCN2A*	c.788C>T	ES（trio）
MO018	Mosaicism	Confirmed diagnostic	Female	8.48	Epilepsy, abnormal muscle tone	*LGI1*	c.377_379del	ES（trio） + SNP array
MO019	Mosaicism	Possibly diagnostic	Male	0.39	Epilepsy	*SCN2A*	c.5533C>T	ES（trio） + SNP array
MO020	Mosaicism	Confirmed diagnostic	Female	0.58	Epilepsy	*SCN1A*	c.827A>C	ES（trio）
MO021	Mosaicism	Confirmed diagnostic	Male	1.5	Epilepsy	*SCN2A*	c.998dupA	ES（trio）
MO022	Mosaicism	Possibly diagnostic	Female	3.1	Epilepsy	*RNF125*	c.485G>A	ES（trio） + SNP array
MO023	Mosaicism	Confirmed diagnostic	Female	Unknown	Epilepsy	*ZBTB18*	c.583C>T	ES（trio）

UPD001	UPD	Confirmed diagnostic	Male	4.8	GDD/ID	*SHOX*	X-chromosome isodisomy	ES（trio）+ SNP array
UPD002	UPD	Confirmed diagnostic	Male	3	GDD/ID	*UBE3A, SNRPN, NDN*	chr15:24,205,790–100,385,477×2	ES（trio）+ SNP array
UPD003	UPD	Confirmed diagnostic	Male	2.6	GDD/ID	*UBE3A, SNRPN, NDN*	chr15:22,382,928–102,398,631×2	ES（trio）+ SNP array
UPD004	UPD	Confirmed diagnostic	Male	2.64	GDD/ID	*UBE3A, SNRPN, NDN*	chr15:22,382,928–102,398,631×2	ES（trio）+ SNP array
UPD005	UPD	Possibly diagnostic	Female	0.3	GDD/ID, facial dysmorphism	*CEMIP*	chr15:81,079,333–102,397,836×2	Targeted panel + SNP array + MLPA
UPD006	UPD	Possibly diagnostic	Male	Unknown	GDD/ID, abnormal muscle tone	*SPRED1, FAM98B, THBS1, SLC24A5*	chr15:38,461,700–59,033,022×2	ES（trio）+ SNP array
UPD007	UPD	Possibly diagnostic	Male	0.9	Abnormal muscle tone	*MEIS2*	chr15:36,595,997–61,045,030×2	Targeted panel + SNP array + MLPA
UPD008	UPD	Possibly diagnostic	Female	8.9	GDD/ID, facial dysmorphism	*NEB*	chr3:123,371,973–174,778,214×2	Targeted panel + SNP array
UPD009	UPD	Possibly diagnostic	Male	12.7	GDD/ID	*GRIN2A, ATF7IP2, EMP2, TEKT5, NUBP1, FAM18*	chr16:86,671–90,160,829×2	ES（trio）+ full-length mitochondrial genome NGS

						A		
MT001	mtDNA heteroplasmy	Confirmed diagnostic	Male	1.3	GDD/ID, microcephaly, abnormal muscle tone	*MT-TL1*	m.3243A>G	ES（trio）+ full-length mitochondrial genome NGS
MT002	mtDNA heteroplasmy	Confirmed diagnostic	Male	10.6	Epilepsy	*MT-TL1*	m.3243A>G	ES（trio）+ full-length mitochondrial genome NGS
MT003	mtDNA heteroplasmy	Confirmed diagnostic	Female	8	Epilepsy	*MT-TL1*	m.3243A>G	ES（trio）+ full-length mitochondrial genome NGS
MT004	mtDNA heteroplasmy	Confirmed diagnostic	Female	11.84	Epilepsy	*MT-TL1*	m.3243A>G	ES（trio）+ full-length mitochondrial genome NGS
MT005	mtDNA heteroplasmy	Confirmed diagnostic	Male	8.01	Epilepsy	*MT-TL1*	m.3243A>G	ES（trio）+ full-length mitochondrial genome NGS

MT006	mtDNA heteroplasmy	Confirmed diagnostic	Male	9	Epilepsy	*MT-TL1*	m.3243A>G	ES（trio）+ full-length mitochondrial genome NGS
MT007	mtDNA heteroplasmy	Confirmed diagnostic	Male	13.8	Epilepsy	*MT-TL1*	m.3243A>G	ES（trio）+ full-length mitochondrial genome NGS
MT008	mtDNA heteroplasmy	Confirmed diagnostic	Male	0.5	Epilepsy, GDD/ID	*MT-ATP6*	m.9185T>C	Targeted panel + full-length mitochondrial genome NGS
MT009	mtDNA heteroplasmy	Confirmed diagnostic	Male	2	Epilepsy, abnormal muscle tone	*MT-ATP6*	m.8993T>C	ES（trio）+ full-length mitochondrial genome NGS
MT010	mtDNA heteroplasmy	Confirmed diagnostic	Female	0.4	Epilepsy	*MT-ATP6*	m.8993T>G	ES（trio）+ full-length mitochondrial genome NGS
MT011	mtDNA heteroplasmy	Confirmed diagnostic	Female	Unknown	Epilepsy	*MT-ATP6*	m.9185T>C	ES（trio）+ full-length mitochondrial genome NGS

MT012	mtDNA heteroplasmy	Confirmed diagnostic	Male	3.1	Movement/ motor or gait disorder	*MT-ATP6*	m.8993T>C	ES（trio）+ full-length mitochondrial genome NGS
MT013	mtDNA heteroplasmy	Confirmed diagnostic	Male	1.6	Abnormal muscle tone, GDD/ID	*MT-ND3*	m.10197G>A	Targeted panel + full-length mitochondrial genome NGS
MT014	mtDNA heteroplasmy	Confirmed diagnostic	Female	2.26	GDD/ID	*MT-ND3*	m.10158T>C	ES（trio）+ full-length mitochondrial genome NGS
MT015	mtDNA heteroplasmy	Confirmed diagnostic	Female	2.81	GDD/ID	*MT-ND5*	m.13513G>A	ES（trio）+ full-length mitochondrial genome NGS
MT016	mtDNA heteroplasmy	Confirmed diagnostic	Female	15	Epilepsy	*MT-ND6*	m.14453G>A	ES（trio）+ full-length mitochondrial genome NGS

The expanded configuration set comprised 200 children with 166 confirmed diagnostic and 34 possibly diagnostic configurations. Candidate-only findings were not included in this table and were retained separately for supplementary descriptive reporting. Each row represents one configuration in one child. ES, exome sequencing; mtDNA, mitochondrial DNA; NGS, next-generation sequencing; UPD, uniparental disomy.

Supplementary Table S2. Clinical Phenotype–Gene Set Mapping and Principal Biological Functions Across Confirmed and Possibly Diagnostic Configurations

**Clinical phenotype group**	**Gene set**	**Principal biological functions/pathways**	**Unique genes, n**	**Children, n**
Confirmed diagnostic configurations (n = 166)				
GDD/ID	*ABCC2, AGAP1, AHI1, ARID1B, ARSA, ARX, ATM, BAG3, BCS1L, CACNA1A, CARKD, CARS2, CASK, CHD2, CSNK2B, CYP2U1, DDC, DMD, ECHS1, ERCC8, ETFDH, FA2H, FARS2, G6PD, GAA, GDI1, GNAO1, HDAC4, HEXA, IQSEC2, KMT2D, L2HGDH, MCPH1, MMACHC, MT-ATP6, MT-ND3, MT-ND5, MT-TL1, MTHFR, NALCN, NDN, NF1, NIPA1, PAH, PANK2, PDHA1, PIGN, PLA2G6, PMM2, POLG, POLR2A, PPP2R1A, RGS10, SCN1A, SCN2A, SCN8A, SHANK3, SHOX, SLC2A1, SLC35A2, SLC6A1, SMC1A, SMN1, SNRPN, SOX5, STS, STXBP1, SYNGAP1, TCF4, TH, TIAL1, TSEN2, UBE3A, VPS13B, WDR62*	Metabolic and mitochondrial processes; synaptic signaling and vesicle trafficking; chromatin and transcriptional regulation	75	82
Epilepsy	*ACADS, ACTB, ACTL6B, ALDH7A1, ARX, ATP7B, BAG3, CARKD, CARS2, CASK, CBLN4, CDH2, CDKL5, CHD2, CSNK2B, CYP27A1, DHX30, DOCK6, FA2H, FKRP, FOXP1, GALC, GCDH, HEXA, HIBCH, HNF1B, KCNQ2, KMT2D, KRAS, LGI1, MC3R, MCPH1, MMACHC, MT-ATP6, MT-ND6, MT-TL1, MTHFR, MUT, NF1, PAH, PCCB, PCDH19, PHACTR1, PIGN, PLA2G6, POLG, POMT2, PPP2R1A, PRF1, PRRT2, PTEN, RARS2, SCN1A, SCN2A, SCN8A, SLC22A5, SLC2A1, SLC35A2, SMC1A, ST3GAL5, STXBP1, TH, TSEN2, TSFM, TYR, UNC13D, WISP3, WWOX, ZBTB18, ZMYND11*	Metabolic and mitochondrial processes; ion-channel and membrane-excitability functions; synaptic signaling and vesicle trafficking	70	94

**Clinical phenotype group**	**Gene set**	**Principal biological functions/pathways**	**Unique genes, n**	**Children, n**
ASD	*CHD2, DMD, ETFDH, GDI1, MMACHC, NALCN, SCN2A, STS*	Metabolic and mitochondrial processes; ion-channel and neuronal signaling functions	8	6
Abnormal muscle tone	*AGAP1, ARID1B, ARSA, FARS2, G6PD, GAA, GNAO1, HDAC4, HIBCH, IQSEC2, KCNQ2, LGI1, MMACHC, MT-ATP6, MT-ND3, MT-TL1, PRRT2, PTEN, SHANK3, SLC2A1, SMN1, TH, TSEN2, ZMYND11*	Metabolic and mitochondrial processes; synaptic and neuronal signaling	24	26
Facial dysmorphism	*AGAP1, DMD, ERCC8, GDI1, HDAC4, KMT2D, NF1, STXBP1, WDR62*	Developmental and chromatin/transcriptional regulation; cytoskeletal/structural functions; synaptic and intracellular trafficking	9	6
Microcephaly	*CASK, DMD, ERCC8, GDI1, IQSEC2, KMT2D, MMACHC, MT-TL1*	Neurodevelopmental and synaptic signaling; chromatin/transcriptional regulation; metabolic processes	8	7
Possibly diagnostic configurations (n = 34)				

Clinical phenotype group	Gene set	Principal biological functions/pathways	Unique genes, n	Children, n
GDD/ID	*ACKR3, ADA2, AKT3, ANK3, ANKH, ATF7IP2, CDKL5, CEMIP, COL4A2, DDX6, DMD, DNAH5, EMP2, FAM18A, FAM20C, FAM98B, GABRG2, GAMT, GLB1, GRIN2A, IL17RA, IL1RAPL1, IQSEC2, ITGB6, KCNT2, KDM5C, MAGEL2, MFSD8, NDP, NEB, NF1, NUBP1, PDGFA, PKP4, SCN8A, SETD5, SGSH, SLC13A5, SLC24A5, SNX14, SPRED1, TEKT5, THBS1, TRRAP, TTBK2, TUBB4A, ZBTB18*	Chromatin and transcriptional regulation; cytoskeletal/structural organization; PI3K–AKT/RAS–MAPK and neuronal signaling	47	22
Epilepsy	*AATF, CDKL5, CIT, COL4A2, FLNA, IQSEC2, ITGB6, KCNT1, KCNT2, LYST, MFSD8, MOGS, NDP, NPRL2, PKP4, PRRT2, RNF125, SCN1A, SCN2A, SCN8A, SCN9A, SLC12A3, SLC13A5, STK11, TRRAP*	Ion-channel and membrane-excitability functions; cytoskeletal/structural organization; synaptic and neuronal signaling	25	17
ASD	*GLB1, SGSH*	Lysosomal and metabolic processes	2	1
Abnormal muscle tone	*AKT3, CDKL5, COL4A2, FAM98B, GLB1, MEIS2, SGSH, SLC13A5, SLC24A5, SPRED1, THBS1, ZBTB18*	Lysosomal/metabolic processes; developmental and transcriptional regulation; ion transport and extracellular-matrix/structural organization	12	6
Facial dysmorphism	*ACKR3, CEMIP, DDX6, KDM5C, NEB, TUBB4A*	Chromatin/transcriptional regulation; cytoskeletal/structural organization; extracellular-matrix and developmental signaling	6	4

Clinical phenotype group	Gene set	Principal biological functions/pathways	Unique genes, n	Children, n
Microcephaly	*AKT3, ZBTB18*	PI3K–AKT-related developmental signaling; transcriptional regulation	2	1

Clinical phenotype groups were not mutually exclusive; therefore, an individual child could contribute to more than one phenotype group, and child counts across rows within each diagnostic block should not be summed. Each configuration corresponded to one child in the expanded configuration set. For multilocus molecular diagnoses, individual component genes were counted separately within each applicable phenotype-specific gene set. Unique-gene counts represent distinct genes observed within each phenotype group and may therefore exceed the number of children. Principal biological functions/pathways were summarized descriptively from established gene/protein functions and represent broad functional patterns within each phenotype-specific gene set; the listed functions/pathways are not intended to be exhaustive, and no formal statistical enrichment analysis was performed. The expanded configuration set comprised 200 children, including 166 with confirmed diagnostic configurations and 34 with possibly diagnostic configurations; candidate-only findings were excluded. Confirmed diagnostic configurations constituted the primary analytical set, whereas possibly diagnostic configurations are presented separately for supplementary descriptive purposes. Patient-level genotypic, diagnostic, and phenotype information for the expanded configuration set is provided in Table S1. GDD/ID, global developmental delay/intellectual disability; ASD, autism spectrum disorder.

Supplementary Table S3. Temporal Sensitivity Analysis of Confirmed Diagnostic Configuration Contribution

Testing era	Total cohort, *N*	Confirmed configurations, *N*	Contribution, *n*/*N* (%)	Adjusted OR (95% CI)	*p* value
2015–2017	495	31	31/495 (6.3%)	Reference	—
2018–2020	735	76	76/735 (10.3%)	1.28 (0.67–2.45)	0.449
2021–2024	932	59	59/932 (6.3%)	0.77 (0.40–1.48)	0.431

The unadjusted distribution of confirmed diagnostic configurations differed across the three testing eras (chi-square p = 0.004). Adjusted ORs and model p values were estimated using logistic regression with confirmed diagnostic configuration status as the outcome, testing era as the primary predictor, and primary testing strategy (trio ES, non-trio ES, or targeted-panel/other testing) as a covariate. The 2015–2017 era was the reference. After adjustment for primary testing strategy, neither later testing era differed significantly from 2015–2017.

Supplementary Table S4. Age at Onset Across Confirmed Diagnostic Configuration Categories

Configuration category	Confirmed configurations, *N*	Age at onset available, *N*	Age at onset, median (IQR), years
Compound heterozygosity	103	101	1.57 (0.40–5.40)
Multilocus molecular diagnosis	24	24	1.65 (0.68–2.88)
Mosaicism	19	18	1.25 (0.59–3.79)
UPD	4	4	2.82 (2.63–3.45)
mtDNA heteroplasmy	16	15	3.10 (1.80–9.80)

Age-at-onset distributions did not differ significantly across the five confirmed diagnostic configuration categories (Kruskal–Wallis $p = 0.160$). Age-at-onset values were unavailable for 2 compound heterozygous, 1 mosaic, and 1 mtDNA configuration. This exploratory cross-category comparison was not included in the Bonferroni correction family.

Supplementary Table S5. Sensitivity Analysis Using the Expanded Configuration Set

Variable	Expanded set (*N* = 200)	Comparison (*N* = 1962)	Crude OR (95% CI)	Adjusted OR (95% CI)	*p* value	Bonferroni-adjusted *p* value
Age at onset, years	2.00 (0.58–5.30) [N = 195]	2.80 (1.00–5.80) [N = 1909]	—	—	0.004	0.031
Male sex	121/200 (60.5%)	1164/1921 (60.6%)	1.00 (0.74–1.34)	0.99 (0.74–1.34)	0.963	1.000
GDD/ID	104/200 (52.0%)	754/1962 (38.4%)	1.74 (1.30–2.32)	1.67 (1.23–2.25)	<0.001	0.008
Epilepsy	111/200 (55.5%)	1338/1962 (68.2%)	0.58 (0.43–0.78)	0.58 (0.43–0.79)	<0.001	0.004
ASD	7/200 (3.5%)	65/1962 (3.3%)	1.06 (0.48–2.34)	0.97 (0.43–2.16)	0.933	1.000
Abnormal muscle tone	32/200 (16.0%)	185/1962 (9.4%)	1.83 (1.22–2.75)	1.80 (1.18–2.73)	0.006	0.050
Facial dysmorphism	10/200 (5.0%)	95/1962 (4.8%)	1.03 (0.53–2.02)	1.08 (0.54–2.13)	0.832	1.000
Microcephaly	8/200 (4.0%)	58/1962 (3.0%)	1.37 (0.64–2.91)	1.28 (0.59–2.79)	0.527	1.000

The expanded configuration set comprised 166 confirmed diagnostic and 34 possibly diagnostic configurations. Age at onset was compared using the Mann–Whitney U test, and no OR or regression effect estimate was reported for this continuous outcome. Crude ORs for binary outcomes were estimated by univariable logistic regression. Adjusted ORs were estimated after controlling for sex, calendar year of testing, and primary testing strategy, with sex excluded as a covariate in the sex model. Bonferroni correction was applied across the eight prespecified clinical comparisons. The Bonferroni-adjusted p value for abnormal muscle tone was approximately 0.050 (0.0501 before rounding) and did not meet the prespecified <0.05 threshold.

Supplementary Table S6. Expanded-Set Compound Heterozygous Configurations

Variable	*n*/*N* (%) or value
Configurations in the expanded set	104
Confirmed diagnostic configurations	103/104 (99.0%)
Possibly diagnostic configurations	1/104 (1.0%)
Two SNV/indel alleles	92/104 (88.5%)
SNV/indel plus CNV <100 kb	11/104 (10.6%)
SNV/indel plus CNV ≥100 kb	1/104 (1.0%)
Any CNV as the second allele	12/104 (11.5%)
Children with available age-at-onset data	102/104 (98.1%)
Neonatal onset (≤30 days)	2/102 (2.0%)
Onset at >30 days to ≤1 year	42/102 (41.2%)
Onset at >1 to ≤3 years	21/102 (20.6%)
Onset at >3 to ≤6 years	18/102 (17.6%)
Onset at >6 to ≤12 years	11/102 (10.8%)
Onset at >12 to ≤18 years	8/102 (7.8%)
Cumulative onset by age 1 year	44/102 (43.1%)
Cumulative onset by age 3 years	65/102 (63.7%)
Cumulative onset by age 6 years	83/102 (81.4%)
Cumulative onset by age 12 years	94/102 (92.2%)

This Table ummarizes the expanded set of 104 compound heterozygous configurations. The main-text confirmed-only analysis comprised 103 configurations, with age-at-onset data available for 101. Percentages below were calculated using either all 104 expanded-set configurations or the 102 children with available age-at-onset data, as appropriate. SNV, single-nucleotide variant. Indel, insertion or deletion. CNV, copy-number variant.

Supplementary Table S7. Expanded-Set Multilocus Molecular Diagnoses

Variable	***n*/*N* (%) or value**
Configurations in the expanded set	48
Confirmed diagnostic configurations	24/48 (50.0%)
Possibly diagnostic configurations	24/48 (50.0%)
At least one de novo variant involved	35/48 (72.9%)
At least one molecular component involving a CNV or exon-level copy-number alteration	16/48 (33.3%)
Children with available age-at-onset data	48/48 (100.0%)
Onset at <0.5 years	5/48 (10.4%)
Onset at 0.5 to <2 years	17/48 (35.4%)
Onset at 2 to <5 years	13/48 (27.1%)
Onset at 5 to <10 years	11/48 (22.9%)
Onset at ≥10 years	2/48 (4.2%)

This Table ummarizes the expanded set of 48 multilocus molecular diagnoses, comprising 24 confirmed diagnostic and 24 possibly diagnostic configurations. The main-text confirmed-only analysis comprised 24 configurations. In that subset, at least one de novo molecular component was identified in 22/24 (91.7%), and at least one component involved a CNV or exon-level copy-number alteration in 12/24 (50.0%). De novo involvement and copy-number components were not mutually exclusive. No single recurrent gene-pair combination predominated. CNV, copy-number variant.

Supplementary Table S8. Expanded-Set Mosaic Findings and Candidate-Only Supplementary Records

Variable	*n*/*N* (%) or value
Expanded configuration set	23
Confirmed diagnostic findings	19/23 (82.6%)
Possibly diagnostic findings	4/23 (17.4%)
Proband/de novo findings	9/23 (39.1%)
Maternal mosaicism	8/23 (34.8%)
Paternal mosaicism	6/23 (26.1%)
Exome sequencing	20/23 (87.0%)
Targeted next-generation sequencing panel	3/23 (13.0%)
Peripheral blood-derived DNA	22/23 (95.7%)
Affected skin or mucosal tissue	1/23 (4.3%)
Sequencing depth available	23/23 (100.0%)
Sequencing depth, median (range)	138.65× (90.68×–184.88×)
Expanded-set proband/de novo VAF, median	28.57%
Maternal mosaicism VAF, median	14.55%
Paternal mosaicism VAF, median	22.00%
Parental mosaicism VAF, median	19.88%
Maternal mosaic findings with VAF <25%	7/8 (87.5%)
Paternal mosaic findings with VAF <25%	4/6 (66.7%)
Parental mosaic findings with VAF <25%	11/14 (78.6%)
Confirmed diagnostic subset	19
Confirmed proband/de novo findings	8/19 (42.1%)
Confirmed maternal mosaic findings	7/19 (36.8%)
Confirmed paternal mosaic findings	4/19 (21.1%)
Confirmed findings assessed by ES	17/19 (89.5%)
Confirmed findings assessed by targeted panel	2/19 (10.5%)
Confirmed findings assessed in peripheral blood	18/19 (94.7%)
Confirmed tissue-restricted finding	1/19 (5.3%)
Confirmed sequencing depth, median (range)	138.42× (90.68×–177.90×)
Confirmed proband/de novo VAF, median	26.79%
Confirmed maternal mosaicism VAF, median	11.00%
Confirmed paternal mosaicism VAF, median	30.00%
Confirmed parental mosaicism VAF, median	20.00%
Confirmed parental mosaic findings with VAF <25%	8/11 (72.7%)
Candidate-only supplementary records	3
Candidate-only origin: proband/de novo	3/3 (100.0%)
Candidate-only VAF values	49.52%, 52.94%, 52.06%
Candidate-only VAF, median	52.06%

The expanded mosaic set comprised 19 confirmed diagnostic and 4 possibly diagnostic findings. Three additional candidate-only findings were excluded from the expanded set and are shown separately for descriptive reporting. Proband/de novo findings included postzygotic variants in the child and de novo findings with mosaic-range VAFs. VAF, variant allele fraction.

Supplementary Table S9. Expanded-Set UPD-Related Configurations

Variable	*n*/*N* (%) or value
UPD configurations in the expanded set	9
Confirmed diagnostic configurations	4/9 (44.4%)
Possibly diagnostic configurations	5/9 (55.6%)
Candidate-only configurations	0/9 (0.0%)
UPD subtype among confirmed diagnostic configurations	
Paternal chromosome 15 UPD consistent with Angelman syndrome	2/4 (50.0%)
Maternal chromosome 15 UPD consistent with Prader–Willi syndrome	1/4 (25.0%)
X-chromosome isodisomy with configuration-specific annotation	1/4 (25.0%)
Chromosomal microarray or SNP-array data documented in available records	8/9 (88.9%)

This Table ummarizes the expanded set of nine UPD-related configurations, including four confirmed diagnostic and five possibly diagnostic configurations. UPD subtype distributions were summarized among the four confirmed diagnostic configurations; therefore, the denominator for the three subtype rows is 4.

Supplementary Table S10. Confirmed Diagnostic mtDNA Heteroplasmy Configurations

Variable	*n*/*N* (%) or value
Confirmed diagnostic mtDNA heteroplasmy configurations	16
Full-length mitochondrial genome next-generation sequencing	16/16 (100.0%)
MT-TL1 m.3243A>G	7/16 (43.8%)
MT-ATP6 variants	5/16 (31.3%)
MT-ND3 variants	2/16 (12.5%)
MT-ND5 variant	1/16 (6.3%)
MT-ND6 variant	1/16 (6.3%)
Maternally inherited findings	11/16 (68.8%)
Not detected in available maternal samples or classified as de novo	5/16 (31.3%)
Paired blood–urine heteroplasmy data available	4/16 (25.0%)
Absolute blood–urine heteroplasmy difference ≤10%	1/4 (25.0%)
Absolute blood–urine heteroplasmy difference >10%	3/4 (75.0%)

Blood–urine heteroplasmy differences were assessed descriptively because paired measurements were available for only four findings. The 10% absolute-difference threshold was used for descriptive summarization and was not considered a validated concordance criterion. mtDNA, mitochondrial DNA.
